# The DAF-16 FOXO Transcription Factor Regulates *natc-1* to Modulate Stress Resistance in *Caenorhabditis elegans*, Linking Insulin/IGF-1 Signaling to Protein N-Terminal Acetylation

**DOI:** 10.1371/journal.pgen.1004703

**Published:** 2014-10-16

**Authors:** Kurt Warnhoff, John T. Murphy, Sandeep Kumar, Daniel L. Schneider, Michelle Peterson, Simon Hsu, James Guthrie, J. David Robertson, Kerry Kornfeld

**Affiliations:** 1 Department of Developmental Biology, Washington University School of Medicine, St. Louis, Missouri, United States of America; 2 Research Reactor Center, University of Missouri, Columbia, Missouri, United States of America; 3 Department of Chemistry, University of Missouri, Columbia, Missouri, United States of America; University of Massachusetts Medical School, United States of America

## Abstract

The insulin/IGF-1 signaling pathway plays a critical role in stress resistance and longevity, but the mechanisms are not fully characterized. To identify genes that mediate stress resistance, we screened for *C. elegans* mutants that can tolerate high levels of dietary zinc. We identified *natc-1*, which encodes an evolutionarily conserved subunit of the N-terminal acetyltransferase C (NAT) complex. N-terminal acetylation is a widespread modification of eukaryotic proteins; however, relatively little is known about the biological functions of NATs. We demonstrated that loss-of-function mutations in *natc-1* cause resistance to a broad-spectrum of physiologic stressors, including multiple metals, heat, and oxidation. The *C. elegans* FOXO transcription factor DAF-16 is a critical target of the insulin/IGF-1 signaling pathway that mediates stress resistance, and DAF-16 is predicted to directly bind the *natc-1* promoter. To characterize the regulation of *natc-1* by DAF-16 and the function of *natc-1* in insulin/IGF-1 signaling, we analyzed molecular and genetic interactions with key components of the insulin/IGF-1 pathway. *natc-1* mRNA levels were repressed by DAF-16 activity, indicating *natc-1* is a physiological target of DAF-16. Genetic studies suggested that *natc-1* functions downstream of *daf-16* to mediate stress resistance and dauer formation. Based on these findings, we hypothesize that *natc-1* is directly regulated by the DAF-16 transcription factor, and *natc-1* is a physiologically significant effector of the insulin/IGF-1 signaling pathway that mediates stress resistance and dauer formation. These studies identify a novel biological function for *natc-1* as a modulator of stress resistance and dauer formation and define a functionally significant downstream effector of the insulin/IGF-1 signaling pathway. Protein N-terminal acetylation mediated by the NatC complex may play an evolutionarily conserved role in regulating stress resistance.

## Author Summary

What are the mechanisms used by animals to cope with stressful environments that inflict damage or restrict essential processes such as growth, development, and reproduction? One strategy is changes in physiology that increase stress resistance, and an extreme version of this strategy is diapause, an alternative developmental state that is enduring and stress resistant. In the nematode *C. elegans*, stress tolerance and entry into a diapause state called dauer larvae are mediated by the conserved insulin/IGF-1 pathway. Specifically, the FOXO transcription factor DAF-16 promotes stress tolerance and dauer larvae development. However, the targets of DAF-16 that mediate these processes remain largely elusive. Using an unbiased forward genetic screen to discover new mediators of stress tolerance, we identified *natc-1*, a novel target of DAF-16 and the insulin/IGF-1 pathway. *natc-1* encodes a conserved subunit of the N-terminal acetyltransferase C (NAT) complex. The NatC complex modifies target proteins by acetylating the N-terminus. We demonstrated that *natc-1* mediates diapause entry and stress tolerance. Furthermore, we elucidated regulation of NatC by demonstrating that *natc-1* is a direct transcriptional target that is repressed by DAF-16. These findings may be relevant to other animals because both the insulin/IGF-1 signaling pathway and the NAT system are conserved during evolution.

## Introduction

The ability to cope with fluctuating environmental stresses is critical for animal survival. Environmental stresses include a wide range of factors such as extremes in temperature, oxidation, and metal availability. A stress response might promote tolerance against a specific challenge or provide broad-spectrum resistance, and a critical question in this field is how specific stress responses mediate resistance to one or more forms of environmental challenge?

The nematode *Caenorhabditis elegans* is an important model system for studies of stress resistance. In response to stresses such as high temperature, low nutrient availability, and high population density, developing larvae will adopt an alternative L3 stage called dauer that is stress resistant [1]. Studies of dauer formation led to the discovery of an insulin/IGF-1 signaling pathway as a critical regulator of this stress response [2]. Loss-of-function mutations in *daf-2* and *age-1* cause dauer constitutive phenotypes, whereas loss-of-function mutations in *daf-16* cause dauer defective phenotypes [1]. *daf-2* encodes an insulin/IGF-1 receptor homolog, and *age-1* encodes a phosphatidylinositol-3-OH kinase catalytic subunit homolog [3], [4]. In addition to mediating a developmental switch in larvae, this pathway functions throughout the life of the animal to mediate stress resistance, since *daf-2* loss-of-function mutations cause increased tolerance to multiple stresses and an extended lifespan [3], [5], [6]. These *daf-2* mutant phenotypes are suppressed by mutations in *daf-16*, indicating that *daf-16* is a major downstream effector of the insulin/IGF-1 signaling pathway that is negatively regulated by *daf-2* activity. *daf-16* encodes a FOXO transcription factor [7], [8]. Because DAF-16 plays a central role in promoting longevity and stress tolerance, a major goal has been to identify and characterize DAF-16 transcriptional targets [9]–[17]. Although several genes have been demonstrated to be directly regulated by DAF-16, the understanding of how DAF-16 target genes mediate stress resistance and longevity remains fragmentary. The insulin/IGF-1 signaling pathway regulates stress tolerance, metabolism, and longevity in multiple species including mammals [18]–[22]. Thus, the identification of physiologically significant DAF-16 targets may suggest strategies for promoting longevity and stress tolerance in *C. elegans* and higher eukaryotes.

The metal zinc is a nutrient that is essential for all organisms and plays many roles in biological systems. Zinc functions in signal transduction pathways and contributes to protein structure and activity [23]–[25]. Zinc deficiency and excess both cause a wide spectrum of defects, demonstrating the importance of zinc homeostasis [26]–[31]. Zinc deficiency appears to be deleterious due to the reduced function of many zinc-requiring proteins and signaling events [25]. The mechanisms underlying excess zinc toxicity are not well characterized; excess zinc may displace other physiological metals or bind to low-affinity sites, leading to altered or decreased protein function [32]. In addition to zinc, several other metals are toxic in excess, including cadmium, nickel, and copper [33]–[35]. To characterize mechanisms of metal stress resistance, we performed a forward genetic screen for mutations that caused resistance to high levels of dietary zinc [36]. We reasoned that tolerance to high zinc could be caused by two general mechanisms: (1) mutations might affect zinc metabolism and reduce the accumulation of toxic zinc, or (2) mutations might cause alterations that promote growth and survival in the presence of high levels of zinc. We identified *haly-1*, which encodes the enzyme histidine ammonia lyase that metabolizes histidine [37]. Loss-of-function mutations in *haly-1* do not affect zinc accumulation but rather cause an increase in histidine levels resulting in increased chelation of zinc and nickel, and chelation by histidine reduces the toxicity of these metals [37]. Notably, *haly-1* mutations have not been demonstrated to cause resistance to other stressors. By contrast, mutations in *daf-2* and *age-1*, members of the insulin/IGF-1 pathway, cause resistance to a broad-spectrum of stressors including the metals cadmium and copper, oxidative stress, and heat stress [5], [35], [38].

Here we describe *natc-1*, a new gene that was discovered in the screen for worms that are resistant to high zinc toxicity, which encodes the *C. elegans* N-α-acetyltransferase 35. N-α-acetyltransferases (NATs) are highly conserved among eukaryotes and function as protein complexes to transfer the acetyl group of acetyl coenzyme A to the α-amino group of the first amino acid of a target protein. *natc-1* encodes an auxiliary subunit of the NatC complex which acetylates proteins that begin with the amino acids Met-Leu, Met-Phe, Met-Ile, and Met-Trp [39], [40]. *natc-1* mutations caused resistance to multiple metals, oxidation, and heat, indicating that *natc-1* modulates broad-spectrum stress resistance, similar to mutations in insulin/IGF-1 signaling genes. Interestingly, the *natc-1* promoter contains an evolutionarily conserved DAF-16 binding site, and DAF-16 binds *natc-1 in vivo* [11], [41], [42]. We demonstrated that *natc-1* is transcriptionally repressed by DAF-16 activity and that *natc-1* interacts with genes in the insulin/IGF-1 signaling pathway to mediate stress resistance and dauer formation. These results indicate that *natc-1* is directly regulated by DAF-16 and functions as a downstream effector of the insulin/IGF-1 signaling pathway. Supporting this model, mutations in *natc-1* that increase stress resistance are epistatic to *daf-16* mutations. These results provide novel insights into the transcriptional regulation of *natc-1* by the insulin/IGF-1 signaling pathway and the biological function of protein N-terminal acetylation in mediating stress resistance. Furthermore, our data elucidate a new mechanism used by the insulin/IGF-1 signaling pathway to mediate stress tolerance and dauer formation.

## Results

### Mutations in *natc-1* caused resistance to high levels of dietary zinc

**Figure 1:**
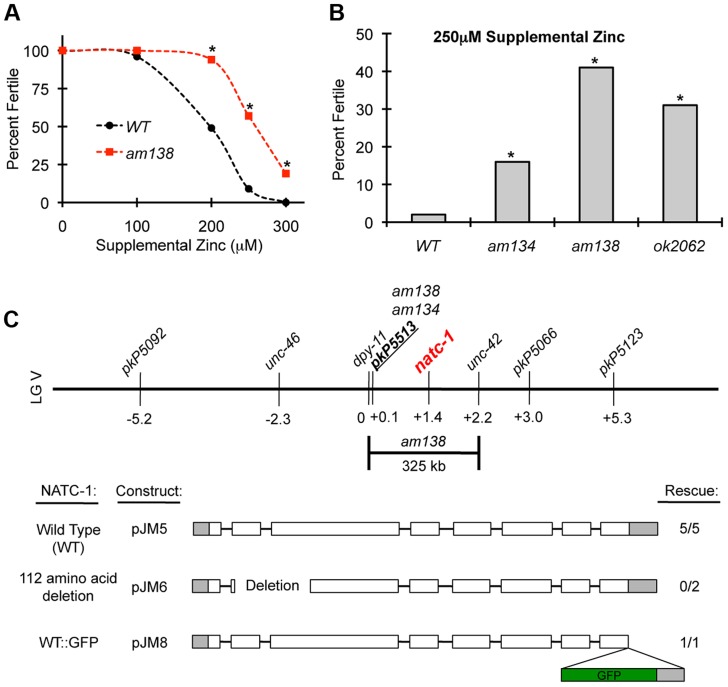
*natc-1* mutations cause resistance to high zinc toxicity. (A) Wild-type (WT) and *natc-1(am138)* embryos were cultured on NAMM medium with *E. coli* as a food source and supplemental zinc (µM). Data points indicate the percent of embryos that developed to adulthood and were fertile based on the generation of at least one progeny (N = 25–226 animals). *natc-1(am138)* animals displayed significant resistance compared to wild-type animals at 200 µM and higher concentrations of supplemental zinc (*, p<0.05). (B) Wild-type, *natc-1(am134)*, *natc-1(am138)*, and *natc-1(ok2062)* embryos were cultured on NAMM supplemented with 250 µM zinc (N = 45–55). All three *natc-1* mutant strains displayed significant resistance to zinc toxicity compared to wild type (*, p<0.05). (C) A genetic map of the center of *C. elegans* linkage group (LG) V. Loci defined by SNPs and mutations that cause visible phenotypes are named above, and positions in map units are shown below. *am134* and *am138* displayed tightest linkage to the SNP *pkP5513* compared to the other SNP markers [36]. Three-factor mapping experiments positioned *am138* between *dpy-11* and *unc-42*, an interval of 325 kb [36] that includes *natc-1* (*T23B12.4*). Transgenic *natc-1(am134)* animals containing extrachromosomal arrays with plasmid pJM5, which encodes wild-type NATC-1, or plasmid pJM6, which encodes NATC-1 with a 112 amino acid deletion from codon 33–144 resulting in a predicted frameshift in the mutated open reading frame, were assayed for zinc resistance. Transgenic *natc-1(am138)* animals containing extrachromosomal arrays with plasmid pJM8, which encodes wild-type NATC-1 fused to green fluorescent protein (GFP), were assayed for zinc resistance. Open boxes indicate exons, and shading indicates untranslated regions. The GFP open reading frame is shaded in green. Rescue indicates the number of independently derived transgenic strains in which transgenic siblings displayed significantly reduced resistance to high zinc compared to nontransgenic siblings and the total number of transgenic strains analyzed (**Figure S1**).

We performed a forward genetic screen to identify mutant strains that are resistant to the growth arrest and lethality caused by high levels of dietary zinc [36]. Nineteen mutations were identified and positioned in the genome using a genome-wide map of single nucleotide polymorphism (SNP) markers [36]. Here we focus on two of these mutations, *am134* and *am138*, that caused significant resistance to dietary zinc toxicity (**Figure 1A,B**). Both mutations displayed tightest linkage to the same SNP, *pkP5513*, positioned at +0.1 map units on chromosome V (**Figure 1C**). Three factor mapping experiments indicated that *am138* is positioned between *dpy-11* and *unc-42*, a 325 kilobase pair interval that contains *pkP5513* (**Figure 1C**) [36].

**Figure 2:**
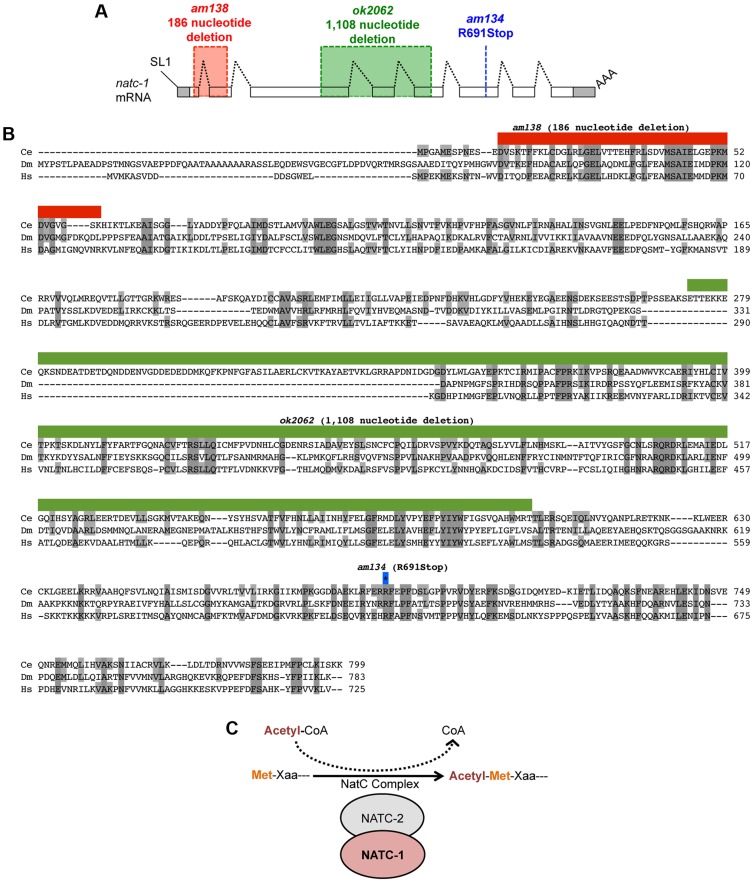
*natc-1* encodes a subunit of the NatC protein acetylation complex. (A) The *natc-1* mRNA structure. Boxes represent exons, and shading indicates untranslated regions. Black dotted lines indicate introns, SL1 (splice leader 1) indicates the 5′ trans-spliced leader sequence, and AAA indicates the 3′ polyA tail. *am138* (red) and *ok2062* (green) are deletion mutations, and *am134* is a nonsense mutation at codon 691 (blue). (B) The predicted *C. elegans* NATC-1 protein is aligned with homologous proteins CG4065 from the insect *Drosophila melanogaster* (Dm) and NAA35 from the vertebrate *Homo sapiens* (Hs) [45]. Shaded amino acids are identical to *C. elegans* NATC-1. Amino acids deleted by *am138* (red) and *ok2062* (green) are shown above. The blue-outlined asterisk indicates the codon mutated in *am134*. (C) A model based on the observations that NATC-1 and *C. elegans* NATC-2 (*B0238.10*) are homologous to an auxiliary and catalytic subunit of the NatC complex, respectively. The NatC complex catalyzes the acetylation of translating proteins at the N-terminus utilizing acetyl-CoA as a substrate. Xaa following Met is typically isoleucine, leucine, tryptophan, or phenylalanine [39], [40].

To identify the gene affected by these mutations, we performed whole genome sequencing using DNA from the *am134* and *am138* mutant strains. Candidate mutations in the mapping interval were identified by comparing the *am134* and *am138* DNA sequence to the wild-type DNA sequence. The *am134* and *am138* strains both contained candidate mutations in the predicted open reading frame *T23B12.4*, suggesting that these mutations caused resistance to zinc toxicity. The predicted *T23B12.4* protein is homologous to human N-α-acetyltransferase 35, an auxiliary subunit of the NatC complex. Thus, we named this gene *natc-1*. The mutation in the *am134* mutant strain, which was induced with the mutagen ENU, is a C to T substitution that changes codon 691 from arginine (CGA) to stop (TGA). This nonsense mutation is predicted to truncate 110 amino acids from the NATC-1 protein (**Figure 2A,B**). The mutation in the *am138* mutant strain, which was induced by ENU mutagenesis, is a 186 base pair deletion that eliminates portions of exons 1 and 2 and all of intron one (**Figure 2A,B**). This deletion eliminates the codons for amino acids 13–59, and the mutated open reading frame is predicted to have a frame shift and encounter a stop at the new codon 15. The loss of coding sequences and early truncation suggest *natc-1(am138)* is likely to be a strong loss-of-function or null allele.

To test the hypothesis that mutations in *natc-1* cause resistance to zinc toxicity, we analyzed the *natc-1(ok2062)* deletion mutation that was generated by the *C. elegans* knock out consortium [43]. *natc-1(ok2062)* is a 1,108 base pair deletion that eliminates a portion of exon 3, intron 3, exon 4, intron 4, and a portion of exon 5 (**Figure 2A,B**). *natc-1(ok2062)* mutant animals displayed significant resistance to zinc toxicity, similar to *natc-1(am134)* and *natc-1(am138)* mutant animals (**Figure 1B**). This result supports the hypothesis that mutations in *natc-1* cause resistance to zinc toxicity.

To independently test the hypothesis that the *am134* mutation in *natc-1* causes resistance to zinc toxicity, we determined whether a wild-type version of *natc-1* could rescue this phenotype. We generated five independently derived transgenic strains containing extrachromosomal arrays with wild-type copies of *natc-1* in the background of *natc-1(am134)*. All the transgenic strains displayed a significant decrease in zinc resistance when compared to their non-transgenic siblings. This is indicative of a more wild-type phenotype and rescue activity (**Figure 1C** **, Figure S1A**). To determine if an intact *natc-1* open reading frame is necessary for rescue activity, we generated transgenic animals containing a *natc-1* locus that encodes a mutant protein with a 112 amino acid deletion in the background of *natc-1(am134)*. These transgenic animals did not display rescue of the mutant phenotype, indicating that the rescue activity of the *natc-1* locus requires an intact open reading frame (**Figure 1C** **, Figure S1B**). Together, these results demonstrated that *natc-1* is the gene affected by *am134* and *am138* (reference allele), and that mutations in *natc-1* caused resistance to zinc toxicity.

### *natc-1* encodes N-α-acetyltransferase 35, an auxiliary subunit of the NatC complex

To characterize the products generated from the *natc-1* locus, we analyzed *natc-1* mRNA. The *C. elegans* expressed sequence tag (EST) project isolated multiple cDNAs corresponding to *natc-1*, and we determined the DNA sequence of three independently derived cDNAs. The cDNA sequences were used to infer the mRNA sequence from exon 3 to the 3′ end, including the position of the polyA tail 330 nucleotides downstream of the TGA stop codon. To characterize the 5′ end of the transcript, we conducted a 5′ RACE experiment that showed the *natc-1* mRNA contains a 22 nucleotide splice leader 1 (SL1) sequence that begins 14 base pairs upstream of the start codon. Together, the analysis of cDNAs and 5′ RACE indicated that the *natc-1* mRNA contains 8 exons and defined the complete predicted open reading frame (**Figure 2A**).

**Figure 3:**
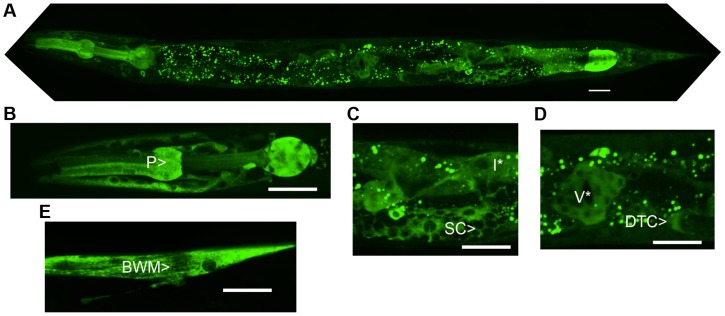
NATC-1 protein is expressed in many cells and tissues and localizes to the cytoplasm. We generated transgenic *natc-1(am138)* animals expressing NATC-1::GFP fusion protein driven by the *natc-1* promoter (WU1449). Confocal fluorescent microscope images of live animals are shown with head on the left and tail on the right. Green represents NATC-1::GFP fusion proteins, except for puncta in intestinal cells visible in panels A, C and D that reflect autofluorescent gut granules. (A) An image of an entire worm. NATC-1::GFP signal is visible in many cells and tissues throughout the animal, and the uniform staining pattern suggests cytoplasmic localization. (B–E) Higher magnification images display fluorescence in the pharynx (P>), sheath cells (SC>), intestinal cells (I*), distal tip cell (DTC>), vulva (V*) and body wall muscle (BWM>). Scale bar is 25 µm for all images.

The predicted NATC-1 protein contains 799 amino acids. To determine the expression pattern and sub-cellular localization of NATC-1, we generated transgenic *natc-1(am138)* animals expressing NATC-1 protein fused to green fluorescent protein (GFP) under the control of the native *natc-1* promoter. Live animals were imaged with confocal fluorescence microscopy, and NATC-1::GFP was detected in a wide range of cells and tissues in a pattern that suggests cytoplasmic localization (**Figure 3**). NATC-1::GFP was detected throughout development from early larval stages through late adulthood. To confirm that the expression pattern of NATC-1::GFP is representative of the expression pattern of endogenous NATC-1, we demonstrated that the extrachromosomal array expressing NATC-1::GFP rescued the *natc-1(am138)* zinc-resistance phenotype (**Figure 1C** **, Figure S1C**).

Comparison of NATC-1 protein sequence to databases using the method of BLAST revealed that NATC-1 is most similar to N-α-acetyltransferase 35 proteins, which are auxiliary subunits of the NatC complex [44]. Figure 2B shows an alignment of *C. elegans* NATC-1 with *Drosophila melanogaster* and human proteins; *C. elegans* NATC-1 is 24% identical to human NAA35, suggesting that it may have similar biochemical functions. NATC-1 is an auxiliary subunit of the NatC complex, and *C. elegans B0238.10* (NATC-2) is the predicted catalytic subunit [45]. The NatC complex catalyzes the acetylation of the N-termini of translating proteins (**Figure 2C**). The NatC complex specifically acetylates translating proteins that begin with Met-Ile, Met-Leu, Met-Trp, or Met-Phe [39], [40]. To identify predicted NatC target proteins, we conducted a bioinformatic analysis using the fully sequenced *C. elegans* genome. Approximately 4,300 proteins have Ile, Leu, Trp, or Phe in amino acid position two. These proteins represent ∼17% of the *C. elegans* proteome and are candidates to be acetylated by the NatC complex.

### The zinc content of *natc-1* mutant animals was similar to wild-type worms

Zinc resistance displayed by *natc-1* mutant animals could be explained by two general models: (1) *natc-1* mutant animals have decreased levels of zinc, perhaps as a result of reduced zinc uptake or increased zinc excretion and (2) *natc-1* mutant animals have normal levels of zinc but increased tolerance to high zinc toxicity. To distinguish between these possibilities, we used inductively coupled plasma mass spectrometry (ICP-MS) to measure total animal zinc content. Synchronized populations of animals were cultured with NAMM, harvested, and analyzed for zinc content. The total animal zinc content of *natc-1(am134)*, *natc-1(am138)*, and *natc-1(ok2062)* mutant animals was not consistently different from wild-type animals when cultured with or without supplemental zinc (**Figure S2**). These results indicate that mutations in *natc-1* cause resistance to zinc toxicity by increasing the ability of the animal to tolerate excess zinc that results from a high zinc diet rather then by reducing zinc accumulation.

### *natc-1(am138)* caused zinc resistance in multiple genetic backgrounds

Mutations in *haly-1* that cause resistance to high zinc toxicity were identified in the same genetic screen as mutations in *natc-1* [37]. The mechanism of action of mutations in *haly-1* appears to be accumulation of histidine, which is hypothesized to reduce high zinc toxicity by chelation of the ion. To determine if mutations in *natc-1* may cause resistance to high zinc toxicity by a similar mechanism, we analyzed *natc-1(am138);haly-1(am132)* double mutant animals. If *natc-1(am138)* and *haly-1(am132)* cause zinc resistance by affecting the same pathway or process, then the resistance to high zinc toxicity phenotypes might not be additive. Interestingly, *natc-1(am138);haly-1(am132)* double mutant animals displayed enhanced resistance to high zinc toxicity compared to *natc-1(am138)* or *haly-1(am132)* single mutant animals (**Figure S3**). This result suggests that resistance to high zinc toxicity caused by *natc-1* mutations may be mechanistically distinct from that caused by *haly-1* mutations.

### Mutations in *natc-1* increased resistance to a wide range of stressors

**Figure 4:**
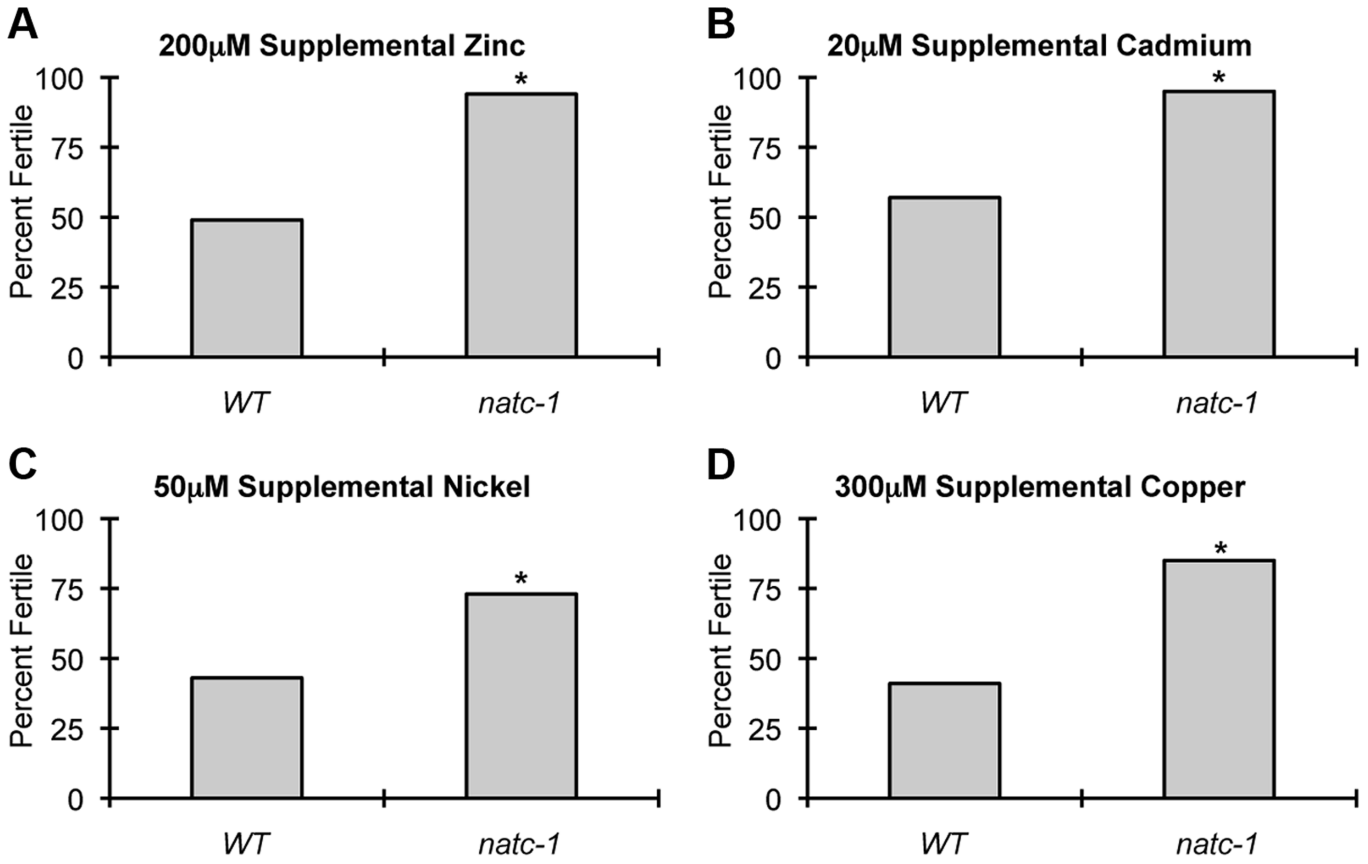
*natc-1* mutations cause resistance to multiple metals. Wild-type (WT) and *natc-1(am138)* embryos were cultured on NAMM supplemented with (A) 200 µM zinc, (B) 20 µM cadmium, (C) 50 µM nickel, or (D) 300 µM copper. Bars display the percentage of fertile adults (N = 37–52). *, p<0.05 compared to WT.

To determine if *natc-1* causes resistance to additional metals, we cultured wild-type animals and *natc-1(am138)* animals on NAMM plates supplemented with cadmium, nickel, or copper. Concentrations that caused ∼50% sterility of wild-type animals were chosen for each metal to maximize the sensitivity of the assay. *natc-1* mutant animals displayed improved growth and development compared to wild-type animals when cultured with 200 µM zinc, 20 µM cadmium, 50 µM nickel, or 300 µM copper (**Figure 4A–D**). These data suggest that mutations in *natc-1* cause resistance to toxicity induced by both physiological and non-physiological metal ions.

**Figure 5:**
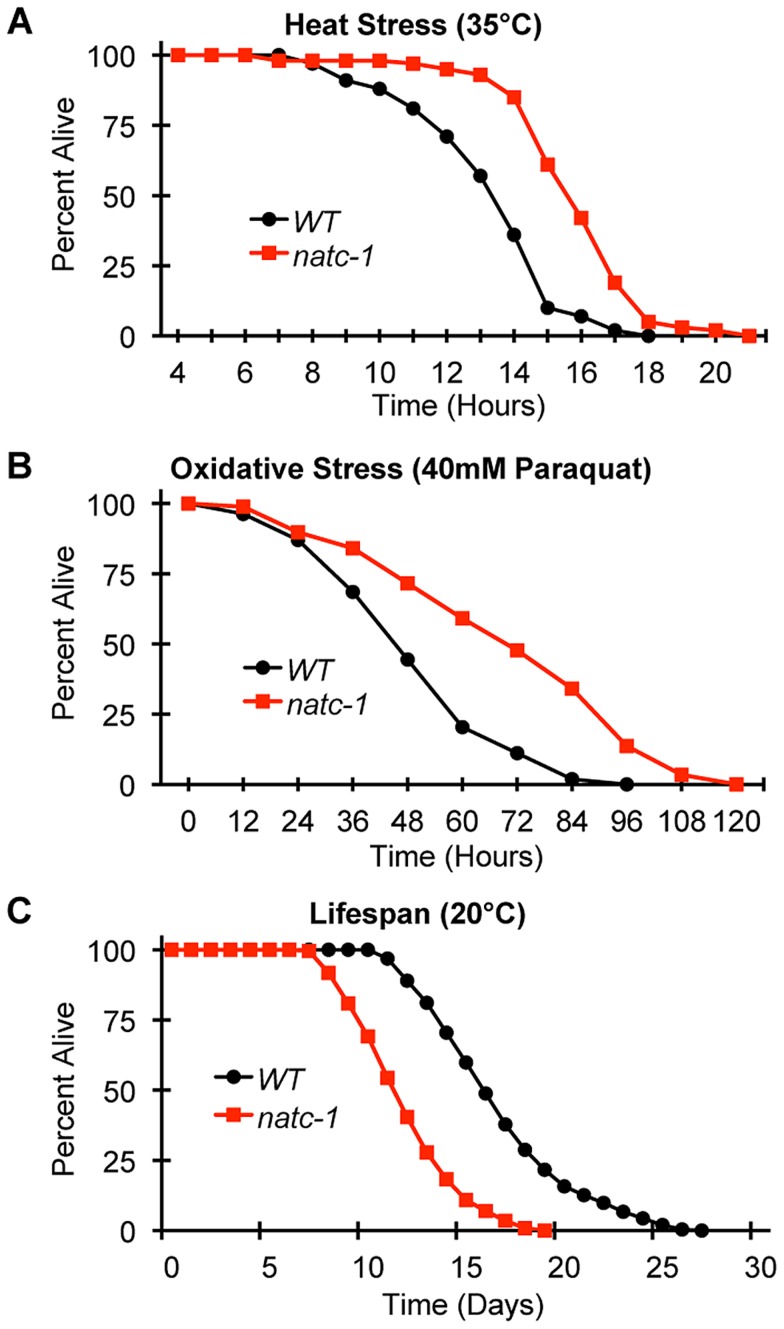
*natc-1* mutations increase resistance to heat and oxidative stress. (A) Wild-type (WT) and *natc-1(am138)* animals were cultured at 15°C on NGM until day 1 of adulthood, shifted to 35°C, and assayed for survival hourly, which involved a brief exposure to room temperature. (B) WT and *natc-1(am138)* animals were cultured at 15°C on NGM until day 3 of adulthood, shifted to NGM containing 40 mM paraquat to induce oxidative stress, and assayed for survival every 12 hours. (C) WT and *natc-1(am138)* animals were cultured at 20°C on NGM and assayed for survival every day. Day 0 is defined as the L4 stage of development (See Table 1 for summary statistics).

To determine if mutations in *natc-1* cause resistance to stressors in addition to metal ions, we analyzed heat stress. Wild-type and *natc-1* mutant animals were cultured at 35°C and survival times were monitored. *natc-1(am138)* animals displayed a significant 19% extension of survival compared to wild-type animals (**Figure 5A** **,** **Table 1**). These data suggest that mutations in *natc-1* cause resistance to heat toxicity. To analyze oxidative stress, we cultured animals with 40 mM paraquat and monitored survival time. *natc-1(am138)* animals displayed a significant 40% extension of survival compared to wild-type animals (**Figure 5B** **,** **Table 1**). Taken together, these data suggest that mutations in *natc-1* cause resistance to a broad-spectrum of stressors, including high levels of multiple metal ions, oxidative damage, and high heat.

**Table 1:** 

Genotype	Assay^1^	Mean Survival^2^	S.D.^2^	Extension of Survival (%)^2^	N^3^	Independent Experiments Performed^3^	Figure^1^
WT	Heat Stress	13.4 (hrs)	2.3	-	58	2	5a,7b
*natc-1(am138)*	Heat Stress	15.9* (hrs)	2.1	19	59	2	5a,7b
*daf-16(mu86)*	Heat Stress	12.7 (hrs)	2.4	−5	64	2	7b
*daf-16(mu86); natc-1(am138)*	Heat Stress	15.6* (hrs)	3.1	16	56	2	7b
WT	Oxidative Stress	51.6 (hrs)	19.3	-	54	4	5b
*natc-1(am138)*	Oxidative Stress	72.3* (hrs)	27.8	40	88	4	5b
WT	Lifespan	15.9 (days)	3.7	-	254	6	5c, S5
*natc-1(am138)*	Lifespan	11.0* (days)	2.6	−31	230	6	5c, S5
*daf-2(e1370)*	Lifespan	34.2* (days)	9.9	116	239	6	S5
*daf-2(e1370); natc-1(am138)*	Lifespan	34.1 (days)	11.5	115	244	6	S5

1 Assay, Figure. Animals were analyzed in standard culture conditions (lifespan) or at 35°C (heat stress) or with 40 mM paraquat (oxidative stress). Data are illustrated in the corresponding Figures.

2 Mean survival, S.D., Extension of Survival. Survival times are presented in hours or days with standard deviation (S.D.). Significant differences are indicated (*, p<0.05). natc-1, daf-16, and daf-2 are compared to WT, daf-16;natc-1 is compared to daf-16, and daf-2;natc-1 is compared to daf-2. Percent extension is compared to WT and can be positive (longer) or negative (shorter).

3 N, independent experiments performed. The total number of animals analyzed and the number of independent experiments performed.

One hypothesis that might explain the stress resistance phenotype is that *natc-1(lf)* mutations stimulate the unfolded protein response. To investigate this hypothesis, we used the method of qRT-PCR to analyze the mRNA levels of the stress-induced genes *hsp-4*, *hsp-6*, and *hsp-16.2*. Wild-type and *natc-1(am138)* animals did not display statistically significant differences in mRNA levels for these genes (p>0.05), suggesting that loss of *natc-1* activity does not stimulate the unfolded protein response. Furthermore, *gst-4* mRNA levels, which are induced by oxidative stress and proteosomal dysfunction [46] were not significantly altered in *natc-1(am138)* mutants compared to wild type (p>0.05).

To determine how *natc-1* activity affects longevity, we analyzed the lifespan of wild-type and *natc-1(am138)* mutant animals. *natc-1(am138)* animals displayed a significant 31% reduction in mean lifespan compared to wild-type animals (**Figure 5C** **,** **Table 1**). These data suggest that *natc-1* is a lifespan assurance gene when animals are cultured at an optimal temperature with abundant food, conditions that minimize stress.

### *natc-1* mRNA appears to be directly repressed by the DAF-16 transcription factor

**Figure 6:**
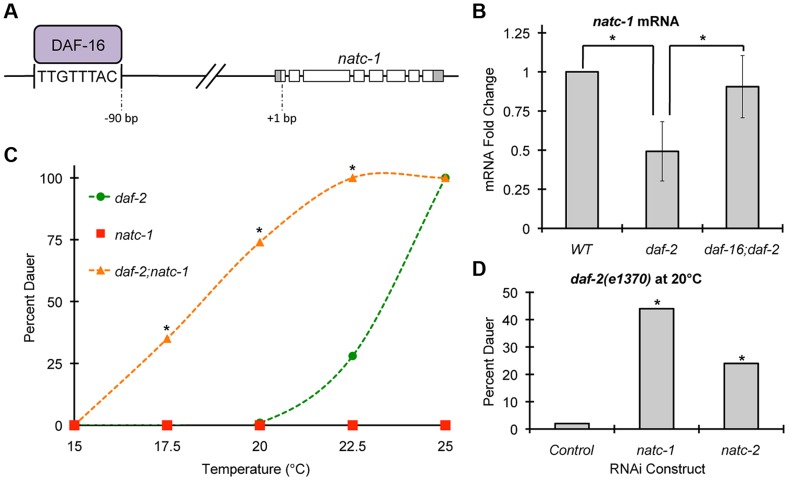
*natc-1* is regulated by *daf-16* and functions in dauer formation. (A) A model of the *natc-1* promoter and open reading frame showing DAF-16 protein binding an evolutionarily conserved DAF-16 binding site positioned 90 base pairs upstream of the *natc-1* start codon [11], [41], [42]. (B) Wild-type, *daf-2(e1370)*, and *daf-16(mu86);daf-2(e1370)* animals were synchronized at the L1 stage of development and cultured for 2 days at 20°C on NGM. For each genotype, mRNA was extracted, analyzed by qRT-PCR, and *natc-1* mRNA levels were normalized to the control gene *rps-23*. Bars show mRNA fold-change values calculated by comparing *daf-2* and *daf-16;daf-2* to WT by the comparative C_T_ method (N = 4–6 biological replicates). Error bars indicate standard deviation. *natc-1* mRNA levels were significantly reduced in *daf-2(e1370)* animals compared to WT but were not significantly different from WT in *daf-16;daf-2* animals (*, p<0.05). The *mtl-1* gene was utilized as a positive control, since it is an established target of DAF-16 [35], and *mtl-1* mRNA levels were significantly increased in *daf-2(e1370)* compared to WT (p<0.05) and this effect was *daf-16* dependent (p<0.05). (C) *daf-2(e1370)*, *natc-1(am138)*, and *daf-2(e1370);natc-1(am138)* embryos were cultured for 4 days on NGM at the indicated temperature and scored as either dauer or non-dauer (N = 200–341). Values for *daf-2;natc-1* were significantly higher than *daf-2* at 17.5°C, 20°C, and 22.5°C (*, p<0.05). (D) *daf-2(e1370)* embryos were cultured on NGM at 20°C, fed *E. coli* expressing dsRNA targeting *natc-1*, *natc-2*, or an empty vector control, and scored as dauer or non-dauer after 4 days (N = 590–627). *natc-1* and *natc-2* RNAi significantly increased dauer formation compared to control (*, p<0.05).

To analyze the regulation of *natc-1*, we used qRT-PCR to monitor the level of *natc-1* mRNA. Because *natc-1(lf)* mutations cause zinc resistance, we examined the transcriptional response to high dietary zinc. *natc-1* mRNA levels were not affected by 200 µM supplemental dietary zinc (p>0.05), suggesting that *natc-1* transcription is not regulated to promote zinc tolerance. To further analyze regulation, we examined the insulin/IGF-1 signaling pathway because it plays a pivotal role in stress resistance in *C. elegans* [2]. *daf-2* encodes the insulin receptor that functions to inhibit dauer formation and stress resistance; *daf-2(e1370)* is a partial loss-of-function mutation that causes a temperature-sensitive dauer constitutive (Daf-c) phenotype in larvae and an increased stress resistance phenotype in adults [6], [35], [38]. *daf-16* encodes a FOXO transcription factor that is a crucial downstream target that is negatively regulated by the DAF-2 pathway; *daf-16(mu86)* is a null mutation that causes a dauer defective (Daf-d) phenotype in larvae and a reduced stress resistance phenotype in adults [7]. Interestingly, Lee *et al.* (2003) used bioinformatic techniques to identify a putative DAF-16 binding site (TTGTTTAC) positioned 90 base pairs upstream of the predicted start codon of the *natc-1* locus [11] (**Figure 6A**). This predicted DAF-16 binding site is evolutionarily conserved in *natc-1* homologues in *Caenorhabditis briggsae* and *Drosophila melanogaster*, suggesting it is functionally important [11]. We analyzed the genomic locus of human NAA35, the homolog of *C. elegans* NATC-1, and identified four predicted DAF-16 binding sites, consistent with the model that these binding sites might be conserved during evolution (**Figure S4**). Furthermore, Gerstein *et al.* (2010) and Riedel *et al.* (2013) used the method of chromatin immunoprecipitation followed by massively parallel DNA sequencing to demonstrate that DAF-16 protein interacts with the *natc-1* locus *in vivo* [41], [42] (G. Ruvkun and C. Riedel, personal communication) (**Figure 6A**).

Based on these observations, we hypothesized that *natc-1* transcription is directly regulated by binding of the DAF-16 transcription factor. According to this model, altering DAF-16 activity is predicted to alter *natc-1* mRNA levels. When cultured in standard laboratory conditions, worms display a low level of DAF-16 activity because the insulin/IGF-1 pathway is strongly activated [47]. Therefore, comparing *daf-16(lf)* animals to control animals would not be highly informative. To test our hypothesis, we analyzed *natc-1* mRNA levels in *daf-2(e1370)* mutant animals, since *daf-2* mutant animals have been demonstrated to have increased DAF-16 nuclear localization and activity [48]. These *daf-2(lf)* phenotypes are similar to the consequences of food deprivation or stress, suggesting *daf-2(lf)* mutant worms have initiated a starvation or stress response [1]. *daf-2* mutant animals displayed a ∼2-fold decrease in *natc-1* mRNA levels compared to wild-type animals (**Figure 6B**). To confirm that this change was statistically significant, we analyzed six independent biological replicates of both wild-type and *daf-2* RNA. These data are consistent with the model that DAF-16 represses transcription of *natc-1*, since DAF-16 activity is increased in *daf-2* mutant animals. To directly test the function of *daf-16*, we analyzed *daf-16;daf-2* double mutant animals; the decrease in *natc-1* mRNA levels was abrogated in these animals, demonstrating that *daf-16* is necessary for the regulation of *natc-1* (**Figure 6B**). To confirm that *daf-16* mutant animals do not contain *daf-16* activity, we analyzed *daf-16* transcript levels by qRT-PCR; *daf-16* transcripts were detected in wild-type animals but were undetectable in the *daf-16;daf-2* double mutant animals. These data suggest that DAF-16 is a transcriptional repressor of *natc-1* and *natc-1* is an effector of the insulin/IGF-1 signaling pathway that functions downstream of DAF-16.

### The NatC complex mediates insulin/IGF-1 signaling during dauer formation

The insulin/IGF-1 signaling pathway mediates entry into an alternative third larval stage called dauer that has distinctive metabolic and developmental features that promote longevity and stress resistance [3], [8], [49]. To test the function of *natc-1* in insulin/IGF-1 signaling, we analyzed dauer larvae formation in *natc-1(am138)* animals. A single mutation in *natc-1* did not cause a Daf-c phenotype (**Figure 6C**). However, *natc-1(am138)* strongly enhanced dauer formation in the *daf-2(e1370)* background, compared to the *daf-2(e1370)* single mutant animals (**Figure 6C**). To determine if the *daf-2(e1370);natc-1(am138)* Daf-c phenotype was *daf-16* dependent, we analyzed *daf-16(mu86);daf-2(e1370);natc-1(am138)* triple mutant animals for dauer formation at 25°C. None of the *daf-16;daf-2;natc-1* triple mutant animals displayed dauer formation (N = 111), indicating that *daf-16* is required for this Daf-c phenotype. Together, these data demonstrated that *natc-1* was necessary to inhibit dauer formation, although the effect was only observed in a sensitive genetic background.

We hypothesized that the enhancement of the Daf-c phenotype caused by a mutation in the *natc-1* auxiliary subunit reflects the reduction or loss of the acetylation activity of the NatC complex. To test this hypothesis, we analyzed the function of the predicted catalytic subunit of the NatC complex, *B0238.10*, which we named *natc-2*. We used the method of feeding RNAi to reduce *natc-1* and *natc-2* activity in a *daf-2(e1370)* mutant background. A significant increase in dauer formation was observed compared to control RNAi (**Figure 6D**). These data indicate that the catalytic subunit encoded by *natc-2* is necessary to inhibit dauer formation, suggesting that the acetylation activity of the NatC complex mediates insulin/IGF-1 signaling.

### *natc-1* functions as a downstream effector of the insulin/IGF-1 signaling pathway to mediate stress resistance

Mutations in the insulin/IGF-1 receptor *daf-2* cause increased longevity, while the *natc-1(am138)* mutation causes a shortened lifespan [50]. To further characterize the genetic interaction between *natc-1* and *daf-2*, we analyzed the lifespan of wild-type, *natc-1(am138)*, *daf-2(e1370)*, and *daf-2(e1370);natc-1(am138)* animals. While *natc-1(am138)* shortens wild-type lifespan, *natc-1(am138)* had no effect on the *daf-2(e1370)* longevity phenotype (**Figure S5,** **Table 1**). This result suggests that *daf-2* activity is necessary for the *natc-1(am138*) mutation to cause a reduction of lifespan.

**Figure 7:**
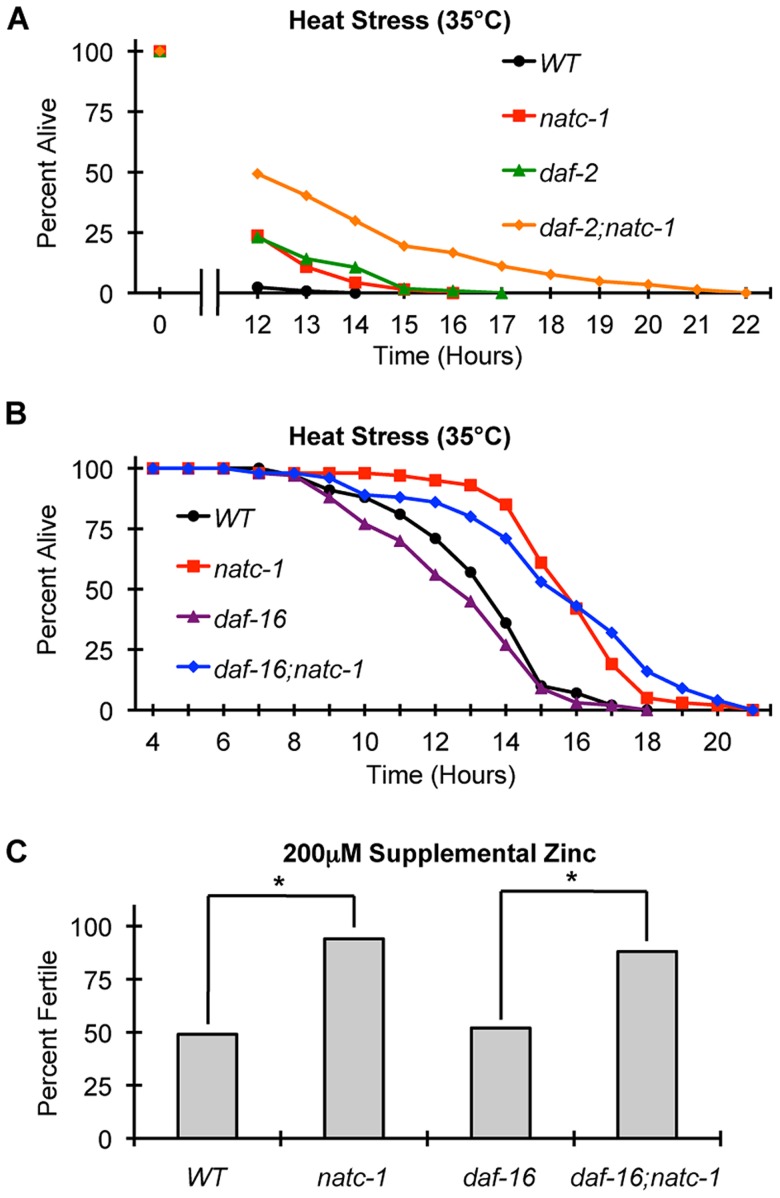
*natc-1* is epistatic to *daf-16* in resistance to heat and zinc stress. (A) Wild-type (WT), *natc-1(am138)*, *daf-2(e1370)*, and *daf-2(e1370);natc-1(am138)* animals were cultured at 15°C on NGM, shifted to 35°C as day 1 adults, and assayed for survival hourly beginning at 12 hours (N = 39–61). (B) Wild-type (WT), *natc-1(am138)*, *daf-16(mu86)*, and *daf-16(mu86);natc-1(am138)* animals were cultured at 15°C on NGM, shifted to 35°C as day 1 adults, and assayed for survival hourly. Summary statistics are presented in Table 1. (C) Embryos were cultured on NAMM with 200 µM supplemental zinc. Bars indicate the percentage of embryos that generated fertile adults. Genotypes were wild type (WT), *natc-1(am138)*, *daf-16(mu86)*, and *daf-16(mu86);natc-1(am138)* (N = 49–54). *daf-16(mu86)* animals were similar to wild-type animals, and *natc-1(am138)* caused significant zinc resistance in wild-type and *daf-16(mu86)* mutant animals (*, p<0.05).

To characterize the role of *natc-1* in stress resistance mediated by the insulin/IGF-1 signaling pathway, we analyzed interactions between *natc-1*, *daf-16*, and *daf-2* in response to heat and high zinc stress. Single mutations of *natc-1* and *daf-2* cause resistance to heat stress, and *daf-2(e1370);natc-1(am138)* double mutant animals displayed enhanced stress resistance compared to either single mutant animal (**Figure 7A**). One interpretation of this additivity is that *natc-1* and *daf-2* function in the same pathway, but neither single mutation maximizes the potential of the pathway to increase stress resistance; this is consistent with the fact that the *daf-2(e1370)* allele causes a partial loss-of-function. The alternative interpretation is that *natc-1* and *daf-2* function in parallel to mediate stress resistance.

Compared to wild-type animals, *daf-16(mu86)* animals displayed a mild sensitivity to heat stress (**Figure 7B** **,** **Table 1**). *daf-16(mu86);natc-1(am138)* double mutant animals displayed heat stress resistance similar to *natc-1* single mutant animals. (**Figure 7B** **,** **Table 1**). These data indicate that *natc-1* is epistatic to *daf-16* with respect to heat stress resistance, consistent with the model that *natc-1* is a downstream effector that is negatively regulated by *daf-16*.

**Figure 8:**
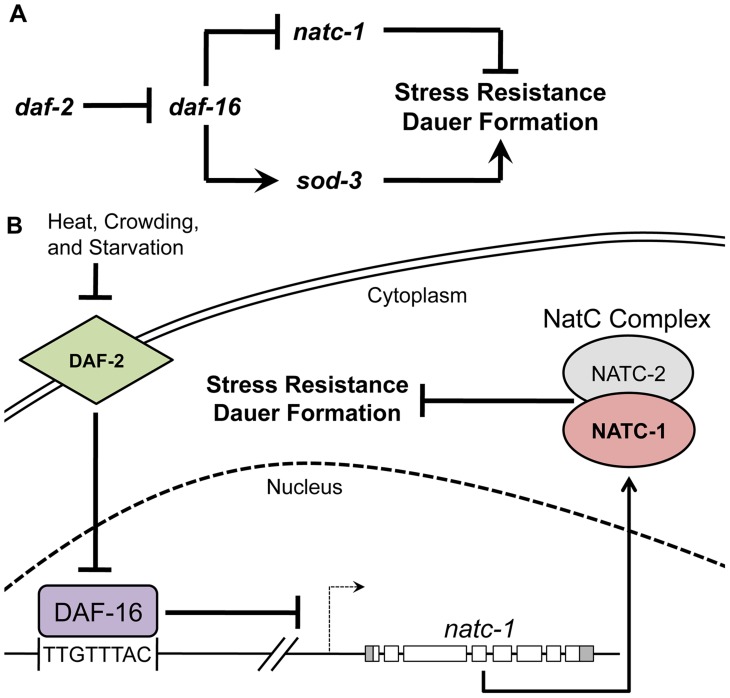
*natc-1* is a physiologically significant downstream effector of the insulin/IGF-1 signaling pathway. (A) A genetic model of *natc-1* function. Arrows and bars indicate positive and negative interactions, respectively, that may be direct or indirect. We propose that *natc-1* negatively regulates stress resistance and dauer formation based on the phenotype of *natc-1(lf)* mutant animals and *natc-1* functions downstream of *daf-16* based on genetic epistasis results and regulation of *natc-1* mRNA levels. Other interactions are based on published studies [1], [54], [88], [89]. In contrast to *sod-3* and other well-characterized DAF-16 target genes that are positively regulated and enhance stress resistance, *natc-1* is negatively regulated and inhibits stress resistance. (B) A combined genetic and molecular model of the function and regulation of *natc-1*. DAF-2 is an insulin/IGF-1 transmembrane receptor that functions through a signaling pathway (not shown) to inhibit the activity of the DAF-16 FOXO transcription factor [90]. We propose DAF-16 directly binds the *natc-1* promoter and represses transcription. *natc-1* transcription and translation promotes activity of the NatC complex, which is predicted to acetylate multiple proteins. However, the role of specific target proteins that modulate stress resistance and dauer larvae formation has not been established.

To further analyze this pathway, we determined if *daf-16* was necessary for *natc-1(am138) to* cause resistance to zinc toxicity. Attempts to analyze *daf-2* and *daf-2;natc-1* mutant animals for resistance to high zinc toxicity were not successful, since supplemental zinc caused a high rate of dauer formation in these mutant animals, precluding an analysis of growth rates (**Figure S6**). *natc-1(am138)* caused similar resistance to high zinc toxicity in both a wild-type and *daf-16(mu86)* background (**Figure 7C**), indicating that *daf-16* function is not necessary for the *natc-1(am138)* zinc-resistance phenotype. These data support the model that *natc-1* functions downstream of *daf-16* to mediate zinc resistance, and together these results suggest that *natc-1* is acting as a key downstream effector of the *C. elegans* insulin/IGF-1 signaling pathway (**Figure 8A,B**).

## Discussion

### *natc-1* is a physiologically relevant effector of the insulin/IGF-1 signaling pathway that is likely to be directly regulated by DAF-16

The DAF-16 FOXO transcription factor is the key downstream target of the insulin/IGF-1 signaling pathway that promotes stress resistance and longevity [5], [35], [50]–. A critical goal in this field is to identify the functionally significant targets of DAF-16, since these genes are hypothesized to mediate stress resistance, nutrient utilization, and aging. A variety of approaches have been used to identify DAF-16 target genes, including bioinformatic, genomic, and reverse genetic techniques [9]–[17]. Lee *et al.* (2003) used the biochemically demonstrated DNA binding sequence of DAF-16 to bioinformatically identify predicted binding sites in the *C. elegans* genome. One of these sites is positioned 90 base pairs upstream of the *natc-1* start codon, suggesting that DAF-16 binds the *natc-1* promoter. A similar FOXO transcription factor binding site is present in the promoters of genes homologous to *natc-1* in *Caenorhabditis briggsae*, *Drosophila melanogaster*, and humans, suggesting that this transcription factor binding site has been conserved during evolution and is functionally significant [11]. Gerstein *et al.* (2010) and Riedel *et al.* (2013) used the technique of chromatin immunoprecipitation followed by DNA sequencing to identify *in vivo* binding sites for DAF-16 (G. Ruvkun and C. Riedel, personal communication) [41], [42]. These studies independently demonstrated that DAF-16 binding is significantly enriched at the *natc-1* locus, consistent with the hypothesis that DAF-16 occupies the predicted binding site.

Here we demonstrated that *natc-1* is transcriptionally repressed by DAF-16. Worms cultured in standard laboratory conditions with abundant food have high activity of the DAF-2 insulin/IGF-1 receptor and low activity of DAF-16. By contrast, *daf-2* loss-of-function mutant animals have high activity of DAF-16 and display enhanced stress resistance and extended longevity. *daf-2(lf)* mutant animals displayed reduced levels of *natc-1* transcripts, and *natc-1* transcripts were restored to WT levels in *daf-16(lf);daf-2(lf)* double mutant animals. These results suggest that *daf-16* activity reduces the level of *natc-1* transcripts, and *daf-2* activity increases the level of *natc-1* transcripts by negatively regulating *daf-16*. Lee *et al.* (2003) used the technique of Northern blotting to examine *natc-1* transcript levels and did not detect a substantial difference between wild-type and *daf-2(e1370)* mutant animals [11]. By contrast, Riedel *et al.* (2013) used the technique of high-throughput sequencing of mRNA (mRNA-Seq) [42] and detected a significant decrease (∼2-fold) in *natc-1* mRNA levels in *daf-2(e1370)* animals compared to wild-type animals (G. Ruvkun and C. Riedel, personal communication). A possible explanation of these findings is that the techniques qRT-PCR and RNA-Seq, which gave similar results, are more quantitative than Northern blotting and were able to detect a small but statistically significant difference in transcript levels. Based on the presence of an evolutionarily conserved DAF-16 binding site in the *natc-1* promoter, the *in vivo* binding of DAF-16 to the *natc-1* locus, and our observed alterations in *natc-1* transcript levels caused by mutations in *daf-2* and *daf-16*, we propose the model that DAF-16 is a direct transcriptional repressor of *natc-1* (**Figure 8B**).

Our genetic analysis showed that *natc-1* inhibits stress resistance and dauer formation and genetically interacts with the insulin/IGF-1 signaling pathway, suggesting that *natc-1* is a physiologically relevant target of DAF-16. These results do not exclude the possibility that *natc-1* might be regulated by an additional pathway(s) in parallel to the insulin/IGF-1 pathway; indeed, the *natc-1* promoter has been reported to interact with multiple transcription factors such as PQM-1, SKN-1, and PHA-4, suggesting there are additional regulatory control mechanisms [41], [53]. *natc-1* mutant animals were discovered in an unbiased forward genetic screen for resistance to the stress of high dietary zinc. Further analysis revealed that *natc-1(lf)* mutations cause resistance to a wide range of stressors, such as heat, oxidation, and multiple metals, similar to *daf-2(lf)* mutations. *daf-2* functions upstream of *daf-16* in a signaling pathway, and *daf-16(lf)* mutations are epistatic to *daf-2(lf)* mutations. By contrast, our model that *natc-1* functions downstream of *daf-16* predicts that the stress resistance caused by *natc-1(lf)* mutations is epistatic to *daf-16(lf)* mutations. Indeed, we observed that the resistance to heat and high levels of dietary zinc caused by *natc-1(lf)* mutations was epistatic to *daf-16(lf)* mutations. Thus, both molecular and genetic studies support the model that *natc-1* is directly targeted by DAF-16 to promote stress resistance. Consistent with this model, we demonstrated that *natc-1* inhibits dauer formation, since *natc-1(lf)* mutations enhanced the dauer constitutive phenotype of *daf-2(lf)* mutations.

Although a variety of DAF-16 target genes have been demonstrated to interact genetically with the insulin/IGF-1 signaling pathway, these target genes are primarily activated by *daf-16*, and loss-of-function of these target genes impairs the stress resistance mediated by *daf-16* activity. For example DAF-16 promotes transcription of the superoxide dismutase *sod-3* [5]. *sod-3* functions to promote resistance to oxidative stress [54]. Thus, *sod-3* represents a class of DAF-16 targets that are transcriptionally activated by DAF-16 and promote stress resistance (**Figure 8A**). To our knowledge, our results with *natc-1* are the first demonstration of a direct DAF-16 target gene that is repressed by DAF-16 to promote stress resistance (**Figure 8A**). Thus, these studies make a novel contribution to the understanding of how the activity of the insulin/IGF-1 signaling pathway mediates stress resistance.

In addition to stress resistance, DAF-16 also promotes longevity [55]. Because mutations in *natc-1* reduce longevity, DAF-16 likely regulates lifespan through a *natc-1*-independent mechanism.

### The NatC complex functions in determining the balance between reproductive growth and stress resistance

N-terminal acetyltransferases (NATs) are multi-subunit enzymes that catalyze the transfer of the acetyl group of acetyl coenzyme A to the α-amino group of the first amino acid of a target protein. N-terminal acetylation is a widespread modification that affects the majority of eukaryotic proteins; for example, ∼80–85% of human proteins are N-terminally acetylated [56]. Eukaryotes possess multiple NAT complexes (NatA-NatE) that vary in subunit composition and substrate specificity [57]. NAT activity is relevant to human health, since mutations in a human NAT gene are associated with Ogden syndrome, an X-linked disorder that is lethal in infancy [58]. Whereas the biochemical activity of NAT enzymes is well characterized, the functional roles of these enzymes are only beginning to be explored.

The NatC complex is comprised of the catalytic subunit Naa30 and the auxiliary subunits Naa35 and Naa38, and it acetylates proteins with the N-terminal sequences Met-Leu, Met-Phe, Met-Ile, and Met-Trp [39], [40], [44], [59], [60]. Genetic studies in yeast revealed NatC complex subunits are necessary for dsRNA virus particle assembly and WT growth rate in media lacking a fermentable carbon source [61], [62]. The *Arabidopsis thaliana* Naa30 homolog was identified in a screen for photosynthesis altered mutants, and Naa30 mutants display decreased chloroplast density and slow growth [63]. The zebrafish Naa35 homolog is the embryonic growth-associated protein (EGAP), and knock down studies with morpholinos suggest that it is necessary for WT growth and development [64]. The rat Naa35 homolog was discovered based on increased expression in healing corneal epithelium, and it is highly expressed in developing rat cornea and skin [65]. In human cells, reducing the levels of NatC complex subunits causes reduced cell viability and p53-dependent apoptosis [66]. These results suggest that the NatC complex functions to promote growth and development in a wide range of organisms.

Our studies of *C. elegans natc-1* make a unique contribution to understanding the biological function of NatC, since these results are the first molecular and genetic characterization of an animal with a NatC subunit mutation. Genetic studies demonstrated that mutations in *natc-1* increased resistance to multiple environmental stressors including excess metal, heat, and oxidation. These results suggest that *natc-1* activity reduces stress resistance. Environments with low food, high population density, and high temperatures promote formation of dauer larvae. Dauers are a stress resistant form that allows animals in unfavorable environmental conditions to suspend development, resist environmental stresses, and be prepared to resume reproductive development when conditions improve [1]. Dauer formation is mediated by the insulin/IGF-1 signaling pathway [1]. The capacity to form dauer larvae highlights the importance of maintaining a balance between promoting growth and reproduction and surviving environmental stressors. In a sensitized genetic background, *natc-1* activity inhibited dauer formation. Because a developmental switch mediates the decision between dauer larvae and larvae destined for reproductive development, these findings indicate that *natc-1* activity promotes a development fate characterized by growth and reproduction. These findings identify novel phenotypes associated with a subunit of the NatC complex.

To determine how the NatC complex might execute these newly discovered functionalities, we bioinformatically identified predicted NatC target proteins in *C. elegans*. NatC might modulate processes such as stress resistance and dauer formation by acetylating groups of proteins with similar functionalities. To investigate this hypothesis, we performed a gene ontology (GO) analysis. Predicted NatC target proteins in *C. elegans* were enriched for 20 functional classes. To determine if NatC has an evolutionarily conserved preference for these functional classes, we used bioinformatics to predict NatC target proteins in humans. GO analyses of human NatC target proteins identified 11 functional classes that were enriched. Electron carrier and oxidoreductase activity were enriched in *C. elegans* and human GO analyses suggesting that the NatC complex may regulate a subset of proteins associated with these functionalities (**Table S1**). We further determined if predicted NatC target proteins were enriched for a specific cellular localization. GO analysis of NatC target proteins identified 4 cellular component terms enriched in *C. elegans* and 18 enriched in humans. NatC target proteins were enriched for mitochondrial localization in both *C. elegans* and humans (**Table S2**). These conserved functionalities and cellular components may inform future experimental efforts aimed at understanding how the NatC complex regulates physiology such as dauer entry and stress tolerance.

To identify protein targets of the NatC complex that might contribute to the mutant phenotype by having altered acetylation in *natc-1(lf*) mutant animals, we identified predicted protein targets that are implicated in zinc metabolism and stress resistance. Published genes implicated in *C. elegans* zinc metabolism include the cation diffusion facilitator (CDF) zinc transporters *cdf-1*, *sur-7*, *cdf-2*, and *ttm-1* [67]–[70], the metallothioneins *mtl-1* and *mtl-*2 [71], and *haly-1* [37]. Of these seven genes, only *cdf-2* is a putative target of the NatC complex. Mutations in *cdf-2* affect zinc accumulation [69], whereas *natc-1(lf)* mutations do not alter zinc accumulation, suggesting that *cdf-2* is not a critical target of the NatC complex in zinc resistance. Rather, we hypothesize that *natc-1* mutations cause high zinc resistance by triggering mechanisms that allow animals to ameliorate the toxicity of high levels of zinc. This hypothesis is supported by the observation that *natc-1* mutations cause resistance to a wide variety of stressors. Genes implicated in stress resistance include those encoding proteins involved in reactive oxygen species metabolism and dauer formation. The NatC complex is predicted to target several proteins that met these criteria; *sod-1*, *sod-2*, and *sod-3* encode superoxide dismutases that increase oxidative stress resistance [54], *mev-1* encodes cytochrome b, a subunit of the mitochondrial respiratory chain complex II, and *frh-1* encodes a frataxin ortholog that promotes the oxidative stress response [72], [73]. Several predicted protein targets are encoded by genes that influence dauer formation; *tph-1* encodes a tryptophan hydroxylase, *daf-36* encodes an oxygenase, and *cyp-35a3* encodes one of 42 cytochrome P450 proteins predicted to be targeted by the NatC complex [17], [74], [75]. Previous studies of stress responses and dauer formation have largely focused on the importance of transcriptional regulation. Our work suggests posttranslational modifications like N-terminal acetylation might also play an important role, and future proteomic analyses may help identify key effectors of stress tolerance and dauer formation.

Our findings are relevant to a general principle that organisms must balance growth and reproduction, which is facilitated by nutrient-rich environments, with stress resistance and quiescence, which are adaptive in nutrient poor and/or high stress environments. Plants that evolved in low-resource, stressful environments share a common set of traits, including relatively low rates of growth, photosynthesis, tissue turnover, and nutrient absorption [76]–[78]. This has been named the “stress resistance syndrome” (SRS), since these plants are resistant to a wide spectrum of physiologic stressors [79]. SRS may represent an adaptive strategy for coping with harsh environmental conditions [76], and this process has interesting analogies to dauer formation in *C. elegans*. Both dauer formation and SRS represent organisms balancing growth and stress tolerance to promote survival. The NatC complex may have an evolutionarily conserved role in mediating this balance between growth and stress tolerance. Consistent with this hypothesis, loss-of-function of the catalytic subunit of the *Arabidopsis* NatC complex causes decreased photosynthetic activity [63], an SRS trait. Additionally, our genetic studies demonstrate that loss-of-function of *natc-1* promotes dauer formation and inhibits reproductive development, a trait analogous to SRS. Therefore, we propose that the NatC complex may have evolutionarily conserved functions in maintaining the balance between promoting growth and reproduction and resisting stressful environmental conditions.

### Regulation of *natc-1*

Given that NatC functions to mediate the balance between growth and stress resistance, which is finely tuned by environmental conditions, it is important to determine how the activity of NatC is regulated. However, little is known about the regulation of these enzymes. In yeast, the *natc-1* homolog (MAK10) protein levels are glucose repressible, but it was not established whether regulation occurs at the level of RNA or protein [61]. Here we demonstrated that *natc-1* is negatively regulated at the level of transcription by DAF-16. The insulin/IGF-1 signaling pathway responds to environmental cues, such as nutrient availability, temperature, and dauer pheromone, by regulating the activity of DAF-16. Therefore, our findings establish a direct link between environmental sensing mediated by the insulin/IGF-1 signaling pathway and protein N-terminal acetylation mediated by NatC. These results make a new contribution to understanding NatC regulation in several ways. First, NAT subunits have not previously been reported to be regulated at the level of transcription. Second, this is a novel demonstration that a NAT complex is regulated by the insulin/IGF-1 signaling pathway, linking an environmental sensing pathway to the regulation of protein N-terminal acetylation (**Figure 8A,B**). NatC complexes appear to have an evolutionarily conserved role in modulating growth and stress resistance, and our findings suggest that they may also have a conserved role in responding to the insulin/IGF-1 signaling pathway.

### Mechanisms of specific and broad-spectrum stress resistance

We have molecularly characterized two genes identified by screening for mutant animals that display increased tolerance to excess dietary zinc [36]: *natc-1* and *haly-1* [37]. These are the only genes that have been demonstrated to cause resistance to excess dietary zinc in an animal, and mutations in these two genes appear to act by very different mechanisms. First, these genes encode proteins with distinct functions. *haly-1* encodes an enzyme that metabolizes histidine, and *haly-1* mutant animals display increased levels of histidine. *natc-1* encodes a subunit of a protein N-terminal acetylation complex, suggesting that protein N-terminal acetylation of many proteins is altered in these mutant animals, although this prediction has not been biochemically tested. Second, the spectrum of stress resistance caused by mutations in these two genes is distinct. Increased histidine appears to chelate and detoxify excess zinc and nickel, but the effect is quite specific since *haly-1* mutant animals are not resistant to the toxicity caused by other metals [37]. By contrast, here we demonstrated that *natc-1* mutations cause broad-spectrum stress resistance, including resistance to multiple metals, heat, and oxidation. Consistent with the model that these genes function by distinct mechanisms, the resistance to excess zinc toxicity caused by mutations of *natc-1* and *haly-1* was additive.

These findings raise a general question about stress resistance; how does a mutation in a single gene such as *natc-1* promote resistance to a broad-spectrum of stresses? (1) One possibility is that the single-gene mutation results in a cascade of events that changes the activity of many proteins in the cell. In this model, each specific change in activity might mediate resistance to only one or a small number of stressors. For example, changes in *haly-1* activity only mediate resistance to zinc and nickel. However, the combination of many different changes in activity could mediate broad-spectrum resistance. This is an attractive model for *daf-2* mutant animals, which display broad-spectrum stress resistance and are documented to have changes in the expression of many genes as a result of the regulation of the DAF-16 transcription factor. This model is also attractive for *natc-1*, since this enzyme is predicted to mediate the N-terminal acetylation of many different proteins. (2) An alternative model is that diverse environmental stresses converge on a single type of important molecular damage. For example, heat, oxidation, and excess metals may all cause toxicity as a result of similar damage, such as protein unfolding. In this model, changing the activity of a single gene might confer broad-spectrum stress resistance by enhancing tolerance to the major form of cellular damage. These two basic models represent extremes of a continuum, and are not mutually exclusive. Our results document that resistance to high zinc toxicity can be increased by mutations that cause specific or broad-spectrum stress resistance, contributing to a conceptual framework for understanding stress resistance.

## Materials and Methods

### General methods and strains

*C. elegans* strains were cultured at 20°C on nematode growth medium (NGM) seeded with *E. coli* OP50 unless otherwise noted [80]. The wild-type *C. elegans* strain and parent of all mutant strains was Bristol N2. The following mutations were used: *daf-16(mu86)* [7] is a strong loss-of-function or null mutation of the DAF-16 forkhead transcription factor; *daf-2(e1370)* [6] is a partial loss-of-function mutation of the DAF-2 insulin/IGF-1 receptor; *haly-1(am132*) [37] is a strong loss-of-function or null mutation of the HALY-1 histidine ammonia lyase. *natc-1(am134)* and *natc-1(am138)* were identified in a genetic screen for resistance to high zinc toxicity [36], backcrossed four times to wild type, and are described here; *natc-1(ok2062)* was obtained from the *C. elegans* knockout consortium [43] and backcrossed four times to wild type. The back crossing procedure replaced ∼94% of the genome of mutant strains with wild-type DNA that has not been exposed to chemical mutagenesis, minimizing background mutations. Double mutant animals were generated by standard methods, and genotypes were confirmed by PCR or DNA sequencing.

### Metal resistance assays

Hermaphrodites were cultured on NGM, and one embryo was transferred to a 35×10 mm Petri dish containing NAMM [36] supplemented with zinc sulfate (ZnSO_4_), cadmium chloride (CdCl_2_), nickel chloride (NiCl_2_), or copper chloride (CuCl_2_) and 5× concentrated *E. coli* OP50 as a food source. Dishes were analyzed daily until progeny were observed or the animal died, except Figure S3 where dishes were analyzed only until day 6. Animals that generated one or more live progeny were scored as “fertile adults.” To determine the metal concentration for these assays, we generated dose response curves of fertility for each metal using wild-type animals and selected the concentration that caused ∼50% of wild-type animals to fail to display fertility (**Figure 4**).

### Determination of zinc content via inductively coupled plasma mass spectrometry

Large populations of animals were cultured on NAMM supplemented with 0 or 200 µM zinc sulfate (ZnSO_4_). The animals were desiccated to determine dry weight, and total zinc content was determined by ICP-MS as described by Murphy *et al.* (2011) [37].

### Imaging of NATC-1::GFP fusion protein

Live transgenic animals were immobilized using levamisole in phosphate buffered saline (PBS) and mounted onto a thin pad of ∼7.5% agarose. More than 100 transgenic animals were analyzed, and representative images are presented. All images were captured on a PerkinElmer spinning disk confocal microscope utilizing Volocity imaging software.

### Heat stress, oxidative stress, and lifespan assays

Gravid adult hermaphrodites were bleached to obtain embryos. Embryos were allowed to hatch in M9 buffer to synchronize at the L1 stage and cultured at 15°C on NGM. For heat stress assays, animals were shifted to 35°C on day 1 of adulthood. Animals were analyzed hourly for spontaneous or provoked motility and pharyngeal pumping; animals displaying none of these traits were scored as dead. Animals were briefly exposed to room temperature (24–25°C) for scoring. For the experiment shown in Figure 7A, animals were cultured continually at 35°C until hourly scoring began at 12 hours; summary statistics were not calculated in this case because some data points were not collected.

For oxidative stress assays, day 3 adults were transferred to NGM dishes supplemented with 40 mM methyl viologen dichloride hydrate (paraquat), fed *E. coli* OP50 and cultured at 20°C. We analyzed day 3 adults to avoid the high frequency of matricidal hatching in response to oxidative stress displayed by younger adults. Animals were scored every 12 hours for survival.

For lifespan assays, L4 animals were cultured on NGM at 20°C (defined as day 0) and fed *E. coli* OP50. Adult hermaphrodites were transferred to fresh Petri dishes every day until the cessation of progeny production and analyzed every day for survival.

In heat stress, oxidative stress and lifespan assays, animals that displayed matricidal hatching or a vulval-bursting phenotype were omitted from the analysis.

### Dauer formation assays

To analyze dauer formation, we transferred embryos to NGM with *E. coli* OP50 at 15–25°C until adult animals began to lay embryos, approximately 3–5 days depending on the temperature. Animals were scored as dauer or non-dauer using a dissecting microscope based on the visible radial constriction phenotype [81]. To determine the effect of zinc, we conducted this assay on NAMM supplemented with zinc sulfate (ZnSO_4_).

To analyze genetic regulation of dauer formation, we performed feeding RNAi as described by Kamath *et al.* (2001) with minor modifications [82]. Briefly, *daf-2(e1370)* P0 hermaphrodites and F1 progeny were incubated at 20°C and continuously fed RNAi expressing bacteria. F1 progeny were scored as dauer or non-dauer after approximately 4 days. We used the empty vector control (L4440) and clones encoding dsRNA corresponding to *T23B12.4* (*natc-1*) and *B0238.10* (*natc-2*) from the Ahringer RNAi Library [83]. The DNA sequence of each clone was confirmed by standard methods.

### Plasmid generation and transgenic rescue

Plasmid pJM5 is pBlueScript SK+ (Stratagene) containing a 3,356 base pair fragment of *C. elegans* genomic DNA from fosmid WRM067bF02 that extends 139 base pairs upstream of the predicted *natc-1* start codon and 395 base pairs downstream of the predicted stop codon. To generate pJM6, we modified pJM5 by digestion with BstEII (New England Biolabs) and religation resulting in a 382 base pair deletion that removes parts of exons 2 and 3 and all of intron 2. The resulting pJM6 mutant open reading frame is predicted to truncate at amino acid 33 in a premature stop codon (TAG). To generate the P*natc-1::NATC-1::GFP::unc-54 3′UTR* translational fusion protein construct (pJM8), we inserted the *natc-1* genomic locus (without the stop codon) into pBlueScript SK+ with the GFP coding sequence and the *unc-54* 3′ UTR. The DNA sequence of each plasmid was confirmed by standard methods.

Transgenic animals were generated by injecting *natc-1(am134)* hermaphrodites with pJM5 or pJM6, and *natc-1(am138)* hermaphrodites with pJM8. All injections were done with the dominant Rol marker pRF4 [84]. We selected independently derived Rol self progeny that transmitted the Rol phenotype. These transgenes formed extrachromosomal arrays, since the Rol phenotype was transmitted to only a sub-set of the self-progeny. To analyze transgenic rescue of the *natc-1(am134)* or *natc-1(am138)* resistance to high zinc toxicity phenotype, we calculated the fraction of transgenic animals on baseline and high zinc concentrations. nonRol animals were presumed to lack the extrachromosomal array and were thus non-transgenic. We defined rescue as a significant decrease in percentage of transgenic animals able to survive (and thus be quantified) on toxic zinc conditions (300 µM supplemental zinc) compared to the baseline zinc concentration (0 µM supplemental zinc), as described by Murphy *et al.* (2011) [37].

### Bioinformatic analysis of targets of the NatC complex

To identify protein targets of the NatC complex, we wrote a custom Perl script that computationally identified predicted NatC targets in *C. elegans* and human protein databases from the National Center for Biotechnology Information (NCBI). Gene ontology (GO) analysis was performed using GOrilla [85] by comparing predicted NatC targets to the entire proteome for each species. We report significant GO functional terms (p<0.001) according to Eden *et al.* (2009) [85].

### *natc-1* mRNA analyses

Three *natc-1* cDNA clones (EST) were obtained from the National Institutes of Genetics, Japan (yk194g4, yk262c3, and yk420a1). We determined the complete sequence of these cDNAs using standard techniques. These data were used to infer the mRNA sequence from exon 3 to the polyA tail attached 330 nucleotides downstream of the TGA stop codon. To experimentally define the 5′ end of the *natc-1* mRNA, we used 5′ RACE System V2.0 (Invitrogen) according to the manufacturer's instructions. These data were used to infer the *natc-1* mRNA sequence from the 22 nucleotide splice leader 1 (SL1) sequence that begins 14 base pairs upstream of the start codon to exon 3.

To generate synchronous populations of worms for RNA extraction, we treated gravid adult hermaphrodite animals with a mixture of bleach and 4M sodium hydroxide (NaOH) and cultured embryos overnight in M9 solution at 20°C, resulting in L1 stage arrest. L1 larvae were transferred to NGM plates at 20°C, fed *E. coli* OP50, and allowed to develop to the L4 stage (approximately 2 days). RNA isolation and cDNA synthesis were performed as described by Davis *et al.* (2009) [86]. Quantitative, real-time PCR was performed using an Applied Biosystems 7900HT Fast Real-Time PCR system and the Applied Biosystems SYBR Green Master Mix. mRNA fold change was calculated using the comparative C_T_ method [87]. Forward and reverse amplification primers were: *rps-23* 5′- aaggctcacattggaactcg and 5′- aggctgcttagcttcgacac; *mtl-1* 5′-ggcttgcaagtgtgactgc and 5′-cctcacagcagtacttctcac; *natc-1* 5′-tcagctttacgggtccaatg and 5′-ccgaaaatgctctgtggttac; *daf-16* 5′- gacggaaggcttaaactcaatg and 5′- gagacagattgtgacggatcg.

### Statistical analysis

All data were analyzed utilizing the two-tailed students t-test of samples with unequal variance unless otherwise specified. For binary data such as dauer entry and fertility, the Chi-squared test was utilized. P-values less than 0.05 were considered statistically significant.

## Supporting Information

Figure S1 **The wild-type** *natc-1* **genomic locus can rescue the** *natc-1(am134)* **zinc-resistance phenotype.** (A) To analyze the activity of the wild-type *natc-1* genomic locus, we generated transgenic *natc-1(am134)* animals that contain an extrachromosomal array composed of the wild-type *natc-1* open reading frame (pJM5) and the transformation marker pRF4 that causes a dominant Rol phenotype. Synchronized populations of worms consisting of transgenic animals that displayed the Rol phenotype and non-transgenic siblings that did not inherit the extrachromosomal array and displayed wild-type movement were allowed to develop on NAMM plates supplemented with 0 or 300 µM supplemental zinc. Bar graphs display the percent of surviving animals that displayed the Rol phenotype. Five independently derived transgenic strains were analyzed, named WU1369, WU1370, WU1371, WU1372, and WU1373. The percent of transgenic animals at 0 µM supplemental zinc varied from 59–75%, which reflects the baseline heritability of the transgenic array that is characteristic of each strain. When cultured with 300 µM supplemental zinc, all five lines displayed a statistically significant reduction in the fraction of transgenic animals, indicating that transgenic animals are less likely to survive compared to non-transgenic siblings in 300 µM supplemental zinc than in 0 µM supplemental zinc. Reduced survival in 300 µM supplemental zinc indicates rescue of the high zinc resistance caused by the *natc-1(am134)*, and we conclude that rescue activity was displayed in all five lines (**Figure 1**) (*, p<0.05). (B) We generated two independently derived transgenic strains (WU1385 and WU1386) using plasmid pJM6, which includes DNA encoding a mutated version of the *natc-1* open reading frame. The strains were analyzed as described above. There was no significant difference between the percent of transgenic animals in 300 µM and 0 µM supplemental zinc, indicating that the mutant NATC-1 protein did not rescue the *natc-1(am134)* phenotype (**Figure 1**). (C) We generated a transgenic strain with an extrachromosomal array composed of pJM8, which encodes wild-type NATC-1 protein fused to GFP, and pRF4 in the background of *natc-1(am138)*. The strain (WU1449) was analyzed as described above. The fraction of transgenic animals was significantly reduced in 300 µM supplemental zinc compared to 0 µM supplemental zinc, indicating that the NATC-1::GFP fusion protein was able to rescue the *natc-1(am138)* mutant phenotype (*, p<0.05). For all three panels, each bar represents the analysis of 3–4 independent biological replicates with an average of ∼40 animals per replicate, and error bars indicate standard deviation.
(TIF)

Figure S2 **Wild-type and** *natc-1* **mutant animals have similar total animal zinc content.** Populations of wild-type, *natc-1(am134)*, *natc-1(am138)*, and *natc-1(ok2062)* animals consisting of a mixture of developmental stages were cultured on NAMM supplemented with 0 or 200 µM zinc. Bars indicate total zinc content determined by ICP-MS and calculated in parts per million (ppm); samples were normalized using dry weight of the worms. *natc-1* mutant strains did not display consistent differences in zinc content compared to wild type.
(TIF)

Figure S3 *natc-1* **and** *haly-1* **mutations cause additive resistance to excess dietary zinc.** Embryos were cultured on NAMM with the 250 µM supplemental zinc. Bars indicate the percentage of embryos that generated fertile adults. Genotypes were wild type (WT), *natc-1(am138)*, *haly-1(am132)*, and *natc-1(am138);haly-1(am132)* (N = 38–53). The resistance to zinc toxicity displayed by *natc-1(am138)* and *haly-1(am132*) single mutant animals was significantly higher than wild-type animals but significantly lower than *natc-1(am138);haly-1(am132*) double mutant animals (*, p<0.05).
(TIF)

Figure S4 **The human NAA35 locus contains predicted DAF-16 binding sites.** A model of the human NAA35 locus on chromosome 12: black bars indicate exons and arrowheads indicate the direction of transcription. Human NAA35 is homologous to *C. elegans* NATC-1. We searched the ∼90 kb locus for predicted DAF-16 binding sites (TTGTTTAC). The positions of four predicted DAF-16 binding sites that were identified are labeled A–D, and the nucleotide sequence and orientation are shown below.
(TIF)

Figure S5 *natc-1(am138)* **did not affect the** *daf-2(e1370)* **lifespan extension.** Wild-type, *natc-1(am138)*, *daf-2(e1370)*, and *daf-2(e1370);natc-1(am138)* animals were cultured at 20°C on NGM and assayed for survival daily. Day 0 is defined as the L4 stage of development. Summary statistics are presented in Table 1.
(TIF)

Figure S6 **Supplemental zinc promotes dauer formation of** *daf-2(e1370)* **and** *daf-2(e1370);natc-1(am138)* **mutant animals.** Wild-type (WT), *natc-1(am138)*, *daf-2(e1370)*, and *daf-2(e1370);natc-1(am138)* hermaphrodites were cultured at 20°C on NGM, and embryos were transferred to NAMM supplemented with 0 or 50 µM zinc and cultured at 17.5°C. After ∼4 days animals were scored as dauer or non-dauer (N = 113–254). When exposed to 50 µM supplemental zinc, *daf-2(e1370)* and *daf-2(e1370);natc-1(am138)* animals displayed increased dauer formation compared to 0 µM supplemental zinc (*, p<0.05).
(TIF)

Table S1 The NatC complex acetylates translating proteins that begin with the amino acids Met-Ile, Met-Leu, Met-Trp, or Met-Phe [39], [40]. We computationally identified all proteins in the *C. elegans* and human proteome that begin with these four amino acid combinations, generating two lists of genes. To identify functions that are enriched in these lists, we performed a functional GO analysis [85]. The columns display statistically significant GO terms (p<0.001), descriptions, and P-values. (1) Electron carrier and (2) oxidoreductase activity were enriched in both *C. elegans* and humans. In addition to electron carrier activity and oxidoreductase activity, we noticed 4 more enriched functional terms in *C. elegans* related to mitochondrial functionality; iron ion binding, oxidoreductase activity (acting on paired donors), heme binding, and tetrapyrrole binding. We also determined that *C. elegans* NatC targets were functionally enriched for 11 terms related to channel/transporter activity; extracellular ligand-gated ion channel activity, ion gated channel activity, gated channel activity, passive transmembrane transporter activity, channel activity, ligand-gated channel activity, ligand-gated ion channel activity, substrate-specific channel activity, ion channel activity, and excitatory extracellular ligand-gated ion channel activity. Furthermore, we identified 2 enriched functional terms in *C. elegans* related to peptidase activity; peptidase regulator activity and peptidase inhibitor activity. In humans we identified 6 functional terms related to peptidase activity; endopeptidase activity, peptidase activity (acting on L-amino acid peptides), serine-type peptidase activity, serine hydrolase activity, serine-type endopeptidase activity, and peptidase activity. Finally in humans we identified 2 functionally enriched terms related to kinase activity; hexokinase activity and glucokinase activity.
(XLSX)

Table S2 The NatC complex acetylates translating proteins that begin with the amino acids Met-Ile, Met-Leu, Met-Trp, or Met-Phe [39], [40]. We computationally identified all proteins in the *C. elegans* and human proteome that begin with these four amino acid combinations, generating two lists of genes. To identify cellular compartments that are enriched in these lists, we performed a cellular component GO analysis [85]. The columns display statistically significant GO terms (p<0.001), descriptions, and P-values. (1) The mitochondrion cellular component was enriched in both *C. elegans* and humans. In addition to the mitochondrion, we noticed 3 more functionally enriched terms in *C. elegans* related to the mitochondria; integral to membrane, intrinsic to membrane, and membrane part. We also identified 9 additional mitochondrial related terms in humans; mitochondrial matrix, mitochondrial part, organelle inner membrane, mitochondrial inner membrane, proton-transporting ATP synthase complex, mitochondrial proton-transporting ATP synthase complex, mitochondrial membrane, mitochondrial membrane part, and mitochondrial crista. We also identified 4 enriched terms in humans related to luminal space; membrane-enclosed lumen, organelle lumen, intracellular organelle lumen, and endoplasmic reticulum lumen. Finally we identified 4 enriched terms in humans related to the extracellular environment; extracellular region part, extracellular space, extracellular region, and collagen.
(XLSX)
