# Supplementary figures and images for: The DAF-16 FOXO Transcription Factor Regulates natc-1 to Modulate Stress Resistance in Caenorhabditis elegans, Linking Insulin/IGF-1 Signaling to Protein N-Terminal Acetylation
Source: PLoS Genet. 2014 Oct 16;10(10):e1004703. doi: 10.1371/journal.pgen.1004703 (PMC4199503; doi:10.1371/journal.pgen.1004703)

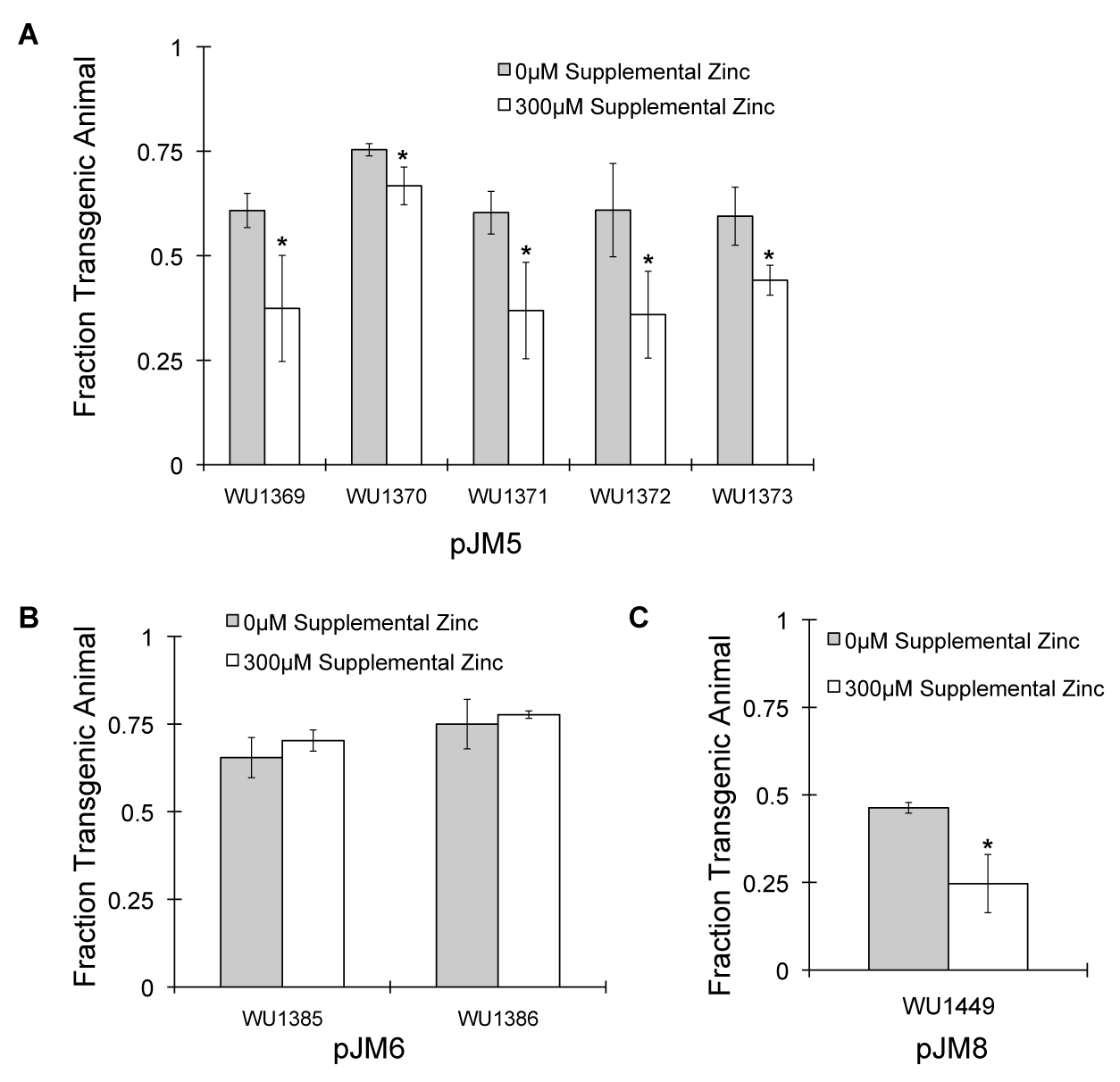

Supplement: Figure S1 — The wild-type natc-1 genomic locus can rescue the natc-1(am134) zinc-resistance phenotype. (A) To analyze the activity of the wild-type natc-1 genomic locus, we generated transgenic natc-1(am134) animals that contain an extrachromosomal array composed of the wild-type natc-1 open reading frame (pJM5) and the transformation marker pRF4 that causes a dominant Rol phenotype. Synchronized populations of worms consisting of transgenic animals that displayed the Rol phenotype and non-transgenic siblings that did not inherit the extrachromosomal array and displayed wild-type movement were allowed to develop on NAMM plates supplemented with 0 or 300 µM supplemental zinc. Bar graphs display the percent of surviving animals that displayed the Rol phenotype. Five independently derived transgenic strains were analyzed, named WU1369, WU1370, WU1371, WU1372, and WU1373. The percent of transgenic animals at 0 µM supplemental zinc varied from 59–75%, which reflects the baseline heritability of the transgenic array that is characteristic of each strain. When cultured with 300 µM supplemental zinc, all five lines displayed a statistically significant reduction in the fraction of transgenic animals, indicating that transgenic animals are less likely to survive compared to non-transgenic siblings in 300 µM supplemental zinc than in 0 µM supplemental zinc. Reduced survival in 300 µM supplemental zinc indicates rescue of the high zinc resistance caused by the natc-1(am134), and we conclude that rescue activity was displayed in all five lines ( Figure 1 ) (*, p<0.05). (B) We generated two independently derived transgenic strains (WU1385 and WU1386) using plasmid pJM6, which includes DNA encoding a mutated version of the natc-1 open reading frame. The strains were analyzed as described above. There was no significant difference between the percent of transgenic animals in 300 µM and 0 µM supplemental zinc, indicating that the mutant NATC-1 protein did not rescue the natc-1(am134) phenotype [file pgen.1004703.s001.tif]

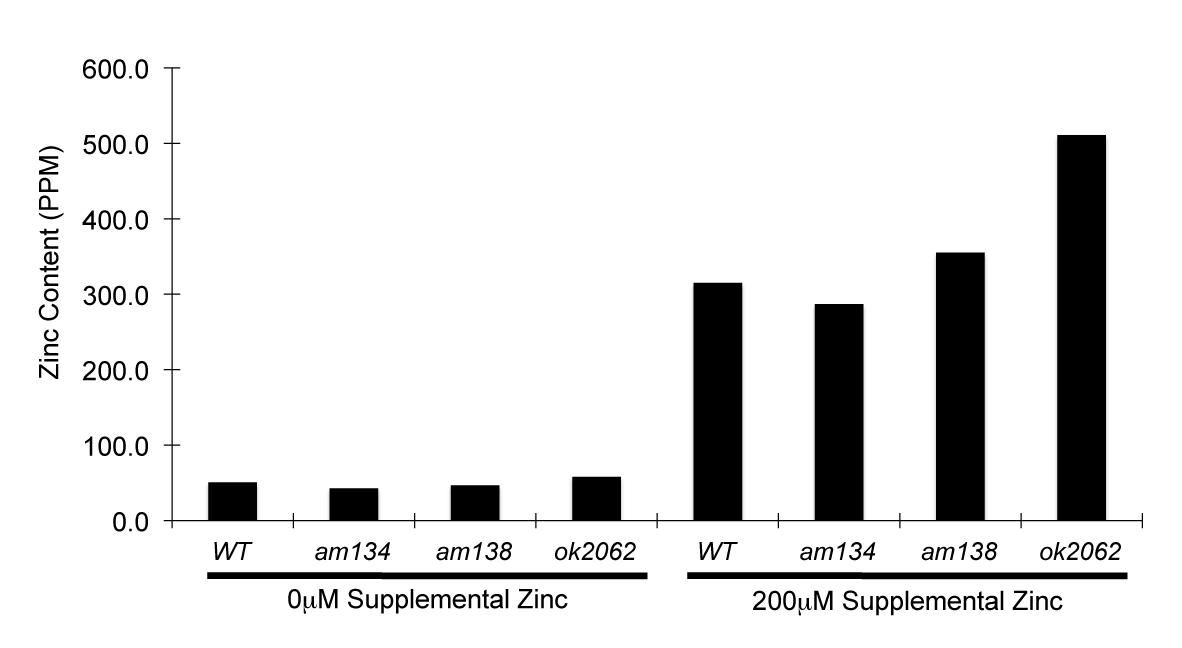

Supplement: Figure S2 — Wild-type and natc-1 mutant animals have similar total animal zinc content. Populations of wild-type, natc-1(am134), natc-1(am138), and natc-1(ok2062) animals consisting of a mixture of developmental stages were cultured on NAMM supplemented with 0 or 200 µM zinc. Bars indicate total zinc content determined by ICP-MS and calculated in parts per million (ppm); samples were normalized using dry weight of the worms. natc-1 mutant strains did not display consistent differences in zinc content compared to wild type. (TIF) [file pgen.1004703.s002.tif]

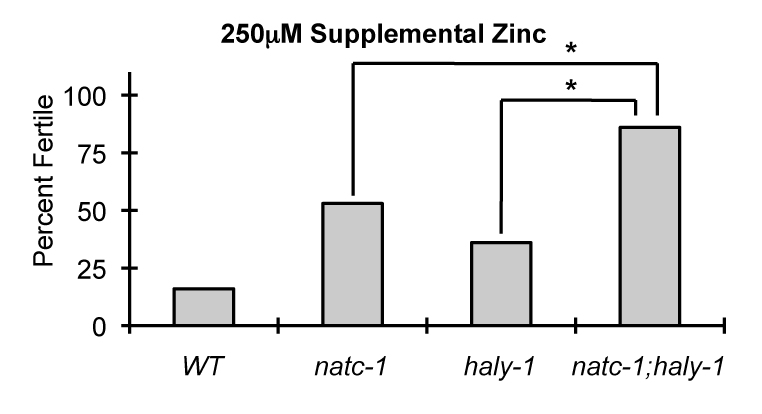

Supplement: Figure S3 — natc-1 and haly-1 mutations cause additive resistance to excess dietary zinc. Embryos were cultured on NAMM with the 250 µM supplemental zinc. Bars indicate the percentage of embryos that generated fertile adults. Genotypes were wild type (WT), natc-1(am138), haly-1(am132), and natc-1(am138);haly-1(am132) (N = 38–53). The resistance to zinc toxicity displayed by natc-1(am138) and haly-1(am132) single mutant animals was significantly higher than wild-type animals but significantly lower than natc-1(am138);haly-1(am132) double mutant animals (*, p<0.05). (TIF) [file pgen.1004703.s003.tif]

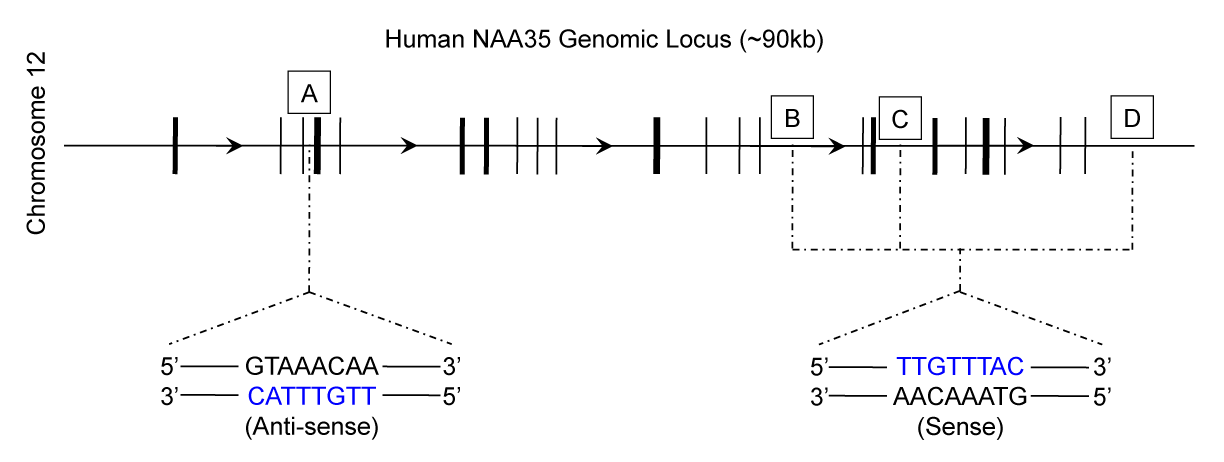

Supplement: Figure S4 — The human NAA35 locus contains predicted DAF-16 binding sites. A model of the human NAA35 locus on chromosome 12: black bars indicate exons and arrowheads indicate the direction of transcription. Human NAA35 is homologous to C. elegans NATC-1. We searched the ∼90 kb locus for predicted DAF-16 binding sites (TTGTTTAC). The positions of four predicted DAF-16 binding sites that were identified are labeled A–D, and the nucleotide sequence and orientation are shown below. (TIF) [file pgen.1004703.s004.tif]

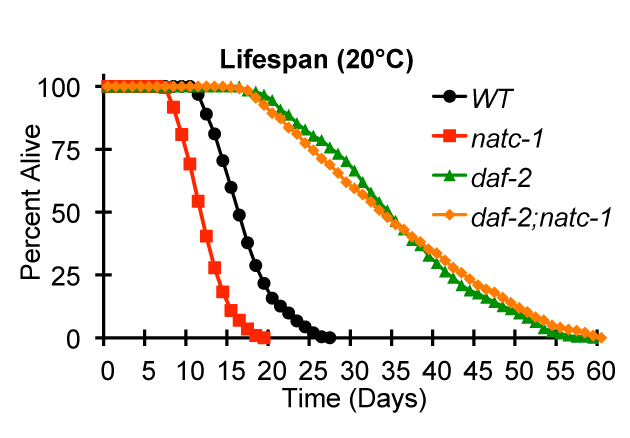

Supplement: Figure S5 — natc-1(am138) did not affect the daf-2(e1370) lifespan extension. Wild-type, natc-1(am138), daf-2(e1370), and daf-2(e1370);natc-1(am138) animals were cultured at 20°C on NGM and assayed for survival daily. Day 0 is defined as the L4 stage of development. Summary statistics are presented in Table 1. (TIF) [file pgen.1004703.s005.tif]

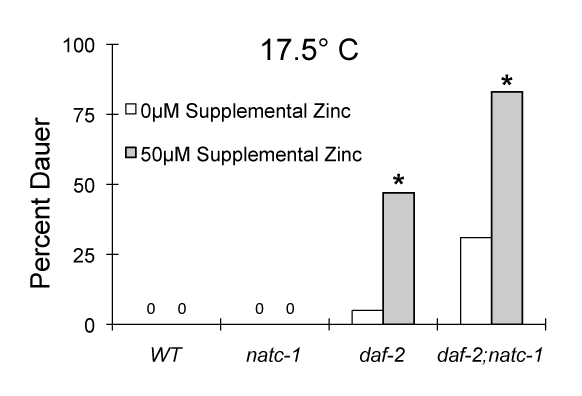

Supplement: Figure S6 — Supplemental zinc promotes dauer formation of daf-2(e1370) and daf-2(e1370);natc-1(am138) mutant animals. Wild-type (WT), natc-1(am138), daf-2(e1370), and daf-2(e1370);natc-1(am138) hermaphrodites were cultured at 20°C on NGM, and embryos were transferred to NAMM supplemented with 0 or 50 µM zinc and cultured at 17.5°C. After ∼4 days animals were scored as dauer or non-dauer (N = 113–254). When exposed to 50 µM supplemental zinc, daf-2(e1370) and daf-2(e1370);natc-1(am138) animals displayed increased dauer formation compared to 0 µM supplemental zinc (*, p<0.05). (TIF) [file pgen.1004703.s006.tif]
